# Semantic associative abilities and executive control functions predict novelty and appropriateness of idea generation

**DOI:** 10.1038/s42003-024-06405-0

**Published:** 2024-06-07

**Authors:** Xueyang Wang, Qunlin Chen, Kaixiang Zhuang, Jingyi Zhang, Robert A. Cortes, Daniel D. Holzman, Li Fan, Cheng Liu, Jiangzhou Sun, Xianrui Li, Yu Li, Qiuyang Feng, Hong Chen, Tingyong Feng, Xu Lei, Qinghua He, Adam E. Green, Jiang Qiu

**Affiliations:** 1 Key Laboratory of Cognition and Personality (SWU), Ministry of Education, Chongqing, China; 2 Faculty of Psychology, Southwest University, Chongqing, China; 3 Department of Psychology, Georgetown University, Washington, DC USA; 4 Southwest University Branch, Collaborative Innovation Center of Assessment Toward Basic Education Quality, Beijing Normal University, Chongqing, China

**Keywords:** Human behaviour, Cognitive neuroscience

## Abstract

Novelty and appropriateness are two fundamental components of creativity. However, the way in which novelty and appropriateness are separated at behavioral and neural levels remains poorly understood. In the present study, we aim to distinguish behavioral and neural bases of novelty and appropriateness of creative idea generation. In alignment with two established theories of creative thinking, which respectively, emphasize semantic association and executive control, behavioral results indicate that novelty relies more on associative abilities, while appropriateness relies more on executive functions. Next, employing a connectome predictive modeling (CPM) approach in resting-state fMRI data, we define two functional network-based models—dominated by interactions within the default network and by interactions within the limbic network—that respectively, predict novelty and appropriateness (i.e., cross-brain prediction). Furthermore, the generalizability and specificity of the two functional connectivity patterns are verified in additional resting-state fMRI and task fMRI. Finally, the two functional connectivity patterns, respectively mediate the relationship between semantic association/executive control and novelty/appropriateness. These findings provide global and predictive distinctions between novelty and appropriateness in creative idea generation.

## Abstract (web summary)

Novelty and appropriateness are two fundamental components of creativity. This study distinguishes novelty and appropriateness of idea generation at the cognitive and neural levels.

## Introduction

Creativity, defined as the ability to generate novel and appropriate ideas or products, plays a vital role in the development of various aspects of society, including scientific^1^ and commercial progress^2^. From blind variation and selective retention^3–5^ until the recently proposed framework named memory in creative ideation^6^, there is extensive evidence suggesting that novelty and appropriateness are the two important factors supporting creative cognition. Although researchers have made efforts to explore theoretically and empirically distinct aspects of creativity^7–17^, the way in which novelty and appropriateness are distinctly instantiated at the behavioral and neural levels requires further investigation. The present research aimed to elucidate the respective behavioral and neural bases of novelty and appropriateness.

Extant evidence indicates that associative abilities^18^ and executive functions^19–21^ are important contributors to creative processes. The association theory of creative thinking emphasizes that creative thinking depends on the ability to generate distant associations^18^. In support of the associative theory, several studies have found that individuals with higher creativity demonstrate stronger associative abilities^22–28^. The application of computational network science tools in recent studies of creativity has made it possible to more accurately quantify associative abilities using semantic distance^29^ (calculated by natural language processing, e.g., Word2vec). Extant research has demonstrated that when the semantic distance between ideas or products is greater, the resulting new ideas or products tend to be more creative^30^. Semantic distance has been more consistently related to novelty than appropriateness. Recent studies have found that novelty is positively correlated with semantic distance, with or without appropriateness as a covariate^31–33^. Therefore, we hypothesize that associative abilities play a greater role in the generation of more novel rather than more appropriate ideas.

On the other hand, the executive theory of creative thinking emphasizes that creative cognition is influenced by top–down execution^19–21^. Studies have examined a range of executive functions in creativity, such as fluid intelligence^20,21,34–36^, working memory^37–40^, cognitive flexibility^19,41^, and inhibitory control^42,43^. Inextricably tied to these “fluid” functions are the representations in memory (i.e., knowledge/experience) on which these functions operate to generate creative ideas by searching, reorganizing, and combining knowledge that is stored in semantic memory^44–49^. Previous research has shown that executive functions support the process of memory retrieval^50–52^. The ability to extract appropriate information from semantic memory may help improve the appropriateness of creative ideas. Therefore, we hypothesize that executive functions play a greater role in the generation of more appropriate rather than more novel ideas. Taken together, a balance of novelty and appropriateness, which may be related to associative abilities and executive functions, respectively, appears requisite for successful creative idea generation^53^.

Functional magnetic resonance imaging (fMRI) studies have identified key regional and connectomic characteristics of creative neurocognition^8,13,54,55^. In general, coupling between and within core networks, including the default mode network (DMN), frontoparietal control network (FPCN), and salience network (SAL)^13^, has been consistently linked to creative ability. Moreover, studies have found that the novelty and appropriateness of ideas are associated with both distinct and shared brain regions. There is evidence that some regional activation (e.g., in the hippocampus and orbitofrontal cortex (OFC)) is associated with appropriate responding, and other regional activation (e.g., in the dorsolateral prefrontal cortex and caudate) is associated with novel responses^56–58^. Specifically, one study used the creative chunk decomposition task, which systematically manipulated novelty and appropriateness through splitting and recombination, and found that the procedural memory system (caudate) is involved in novelty processing, while the episodic memory system (hippocampus) is involved in appropriateness processing^58^. Another study used a riddle-based task and found that the dorsolateral prefrontal cortex was involved during novelty processing, whereas the hippocampus, amygdala, and OFC were involved during appropriateness processing; additionally, both novelty and appropriateness processing were mediated by the temporoparietal junction^57^. Recently, a study directly manipulated the novelty and appropriateness of objects, placing the same object in three different conditions to study how novelty and appropriateness features are processed: (1) familiar and useful, (2) novel and useful, and (3) novel and useless. By comparing different conditions, the study found novelty and appropriateness were both related to the middle temporal gyrus and medial temporal lobe. Rather than examining regional markers, a whole-brain connectomic approach may afford the potential to globally and predictively distinguish the respective neural implementations of novelty and appropriateness^8,59–61^. In particular, CPM^62^ may allow data-driven isolation of the neural network signatures of novelty and appropriateness, and prediction of these two aspects of creativity from an individual’s brain connectivity.

In the present study, we aimed to distinguish the novelty and appropriateness of creative idea generation at both the behavioral and neural levels. First, at the behavioral level, we tested a hypothesized dichotomy—based on the associative and executive theories of creativity—that the novelty of creative idea generation relies more on associative abilities and the appropriateness of creative idea generation relies more on executive functions. Next, in a resting-state fMRI (rs-fMRI) dataset, we defined functional network-based models for novelty and appropriateness that achieved cross-brain prediction of novelty and appropriateness. Furthermore, the generalizability and specificity of the two functional connectivity patterns were verified in additional rs-fMRI and task-fMRI analyses. Finally, the two functional connectivity patterns (respectively) mediated the relationship between semantic association/executive control and novelty/appropriateness.

## Results

### Novelty and appropriateness are dissociated by associative abilities and executive functions

**Fig. 1:**
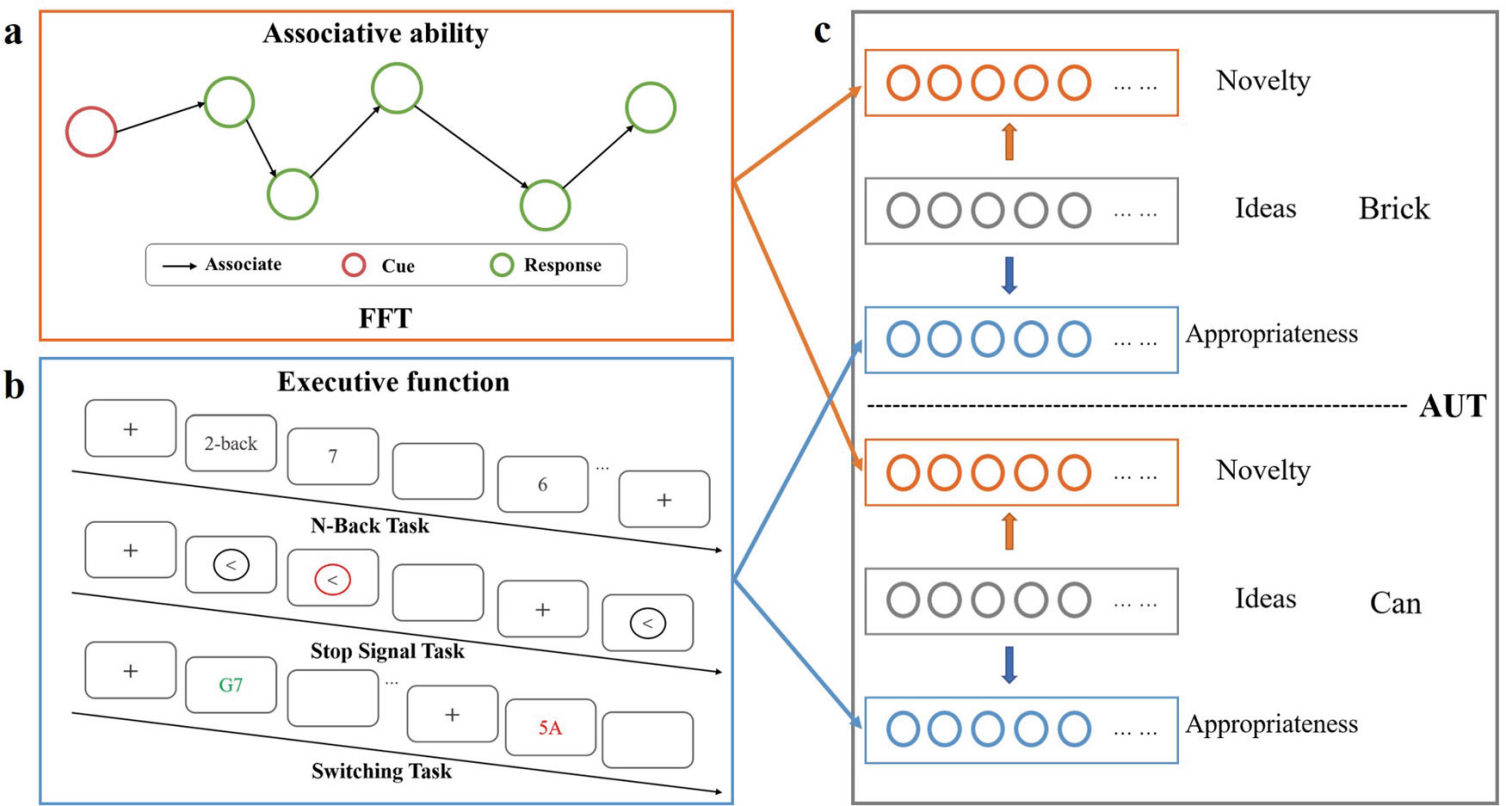
Estimation of associative abilities, executive functions, and creativity. **a** One example item and schematic of FFT. The red circle represents the cue word; the green circles represent the response words; and the black arrow represents the associative process. FFT was used to measure associative abilities. **b** Schematic of n-back task (top), stop signal task (middle), and switching task (also called number–letter category switching task, bottom). These tasks were used to measure executive functions. **c** Schematic of AUT. Brick and Can are two items of AUT. Gray circles represent the ideas generated by participants based on each item. Orange circles represent the novelty of the ideas. Blue circles represent the appropriateness of the ideas. We hypothesized that associative abilities are more responsible for the novelty of the creative ideas generated and executive functions is more responsible for the appropriateness of the ideas generated. FFT forward flow test, AUT alternative uses task.

Novelty and appropriateness are two primary aspects of evaluating creativity, and they may rely on different cognitive abilities. Here, we hypothesize that the generation of more novel ideas is associated with higher associative abilities, while the generation of more appropriate ideas is associated with higher executive functions. We tested these hypotheses using data (*n* = 1509, 1014 females, age *M* = 21.1, s.d. = 0.97) from the Behavioral Brain Research Project of Chinese Personality (BBP)—associative abilities was measured using the forward flow test (FFT) (Fig. 1a); distinct components of executive functions were measured using the n-back task (measure working memory), the stop signal task (measure inhibitory control) and the number–letter category switching task (measure cognitive flexibility) (Fig. 1b). Novelty and appropriateness were measured using two alternative uses task (AUT) items (Brick and Can, Fig. 1c).

Four trained raters evaluated the two AUT items to obtain scores of novelty and appropriateness of every participant; the higher the score, the better the novelty or appropriateness of the participant. The mean standardized reaction time (RT) of three executive function tasks was used as an indicator to represent the ability of executive functions; the shorter the RT, the better the executive functions. Meanwhile, the mean standardized accuracy (ACC) of three executive function tasks was controlled in the general linear model^40^, due to the speed-accuracy trade-off. In addition, the standardized semantic distance^9,25^ of FFT represented the associative abilities; the higher the score, the better the associative abilities.

**Fig. 2:**
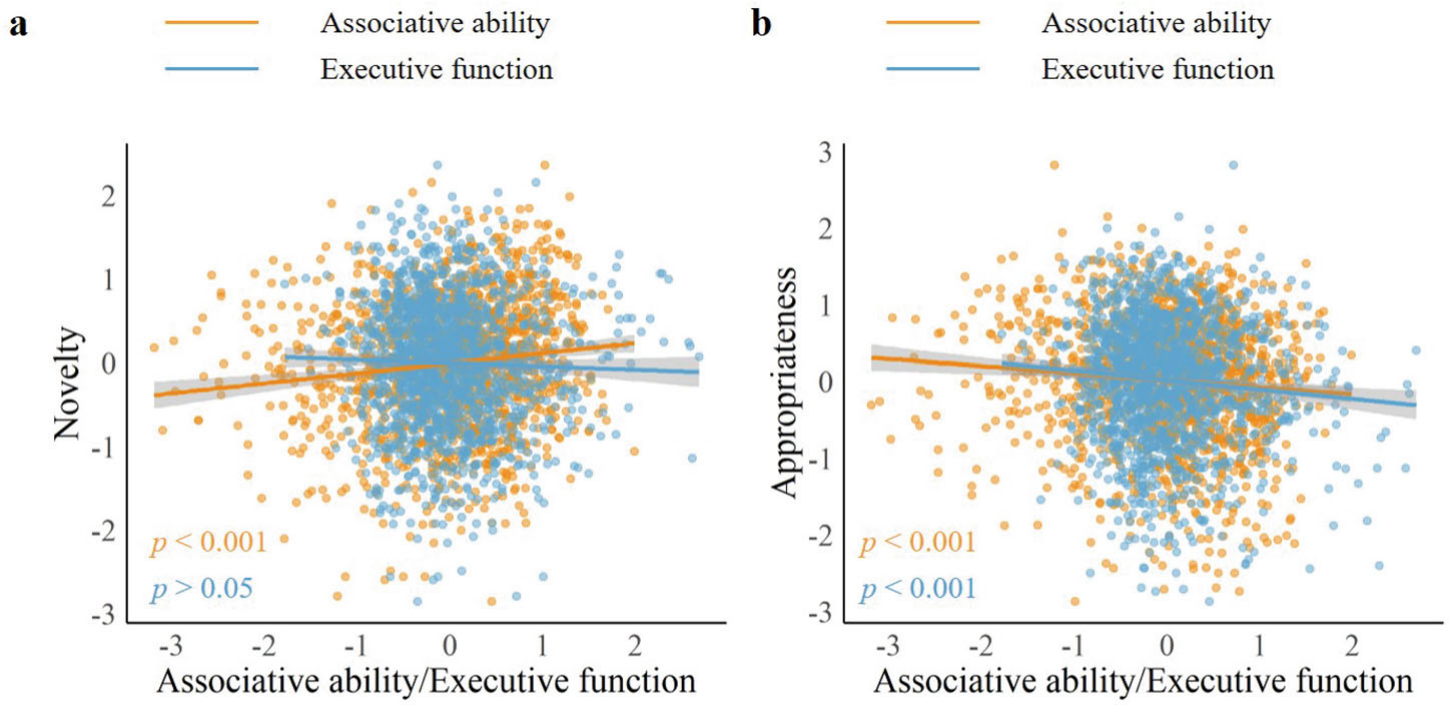
Results of general linear models predicting novelty and appropriateness. **a** The results indicated that novelty was only predicted by associative abilities (orange) but not executive functions (blue). **b** The result showed that the faster the RT of executive functions the higher the appropriateness, which indicated that appropriateness was positively predicted by executive functions (blue). Another result showed that appropriateness was negatively predicted by associative abilities (orange). Novelty = the standardized score of novelty, appropriateness = the standardized score of appropriateness; associative abilities = the standardized semantic distance of FFT, executive functions = the mean standardized RT of three executive functions tasks. Source data are provided as a Supplementary Data file.

To explore our hypothesis, we investigated the effects of associative abilities and executive functions on novelty and appropriateness. The first model included associative abilities and executive functions as predictors (Table S1, Model 1). General linear models (GLM) indicated that novelty was predicted by associative abilities, such that higher associative abilities were positively associated with higher novelty ratings (*n*  =  1509, *β*  =  0.12, s.e.  =  0.03, *t*_1504_  =  4.71, Bonferroni corrected *p*  < 0.001, Fig. 2a orange); on the contrary, there was no significant association between novelty and executive functions (*n*  =  1509, *β*  =  −0.02, s.e.  =  0.03, *t*_1504_  =  −0.69, Bonferroni corrected *p*  > 0.05, Fig. 2a blue). The interaction between associative abilities and executive functions on novelty was not significant (*n*  =  1509, *β*  =  −0.01, s.e.  =  0.04, *t*_1504_  =  −0.24, Bonferroni corrected *p*  > 0.05, Table S1). On the other hand, GLM indicated that appropriateness was predicted by executive functions, such that higher executive functions (shorter RT) were associated with higher appropriateness ratings (*n*  =  1509, *β*  =  −0.09, s.e.  =  0.03, *t*_1504_  =  −3.35, Bonferroni corrected *p*  < 0.001, Fig. 2b blue). Appropriateness was also predicted by associative abilities, in the opposite direction of executive functions (*n*  =  1509, *β*  =  −0.09, s.e.  =  0.04, *t*_1504_  =  −3.63, Bonferroni corrected *p*  < 0.001, Fig. 2b orange). The interaction between associative abilities and executive functions on appropriateness was also not significant (*n*  =  1509, *β*  =  0.03, s.e.  =  0.04, *t*_1504_  =  1.02, Bonferroni corrected *p*  > 0.05, Table S1). Moreover, these results remain consistent after controlling for gender, age, and handedness (Table S1, Model 2). Therefore, these results together confirmed our hypotheses that associative abilities and executive functions act independently on novelty and appropriateness; specifically that the generation of more novel ideas relied more on associative abilities, while the generation of more appropriate ideas relied more on executive functions.

### Two distinct connectome predictive models predict novelty and appropriateness across brains

**Fig. 3:**
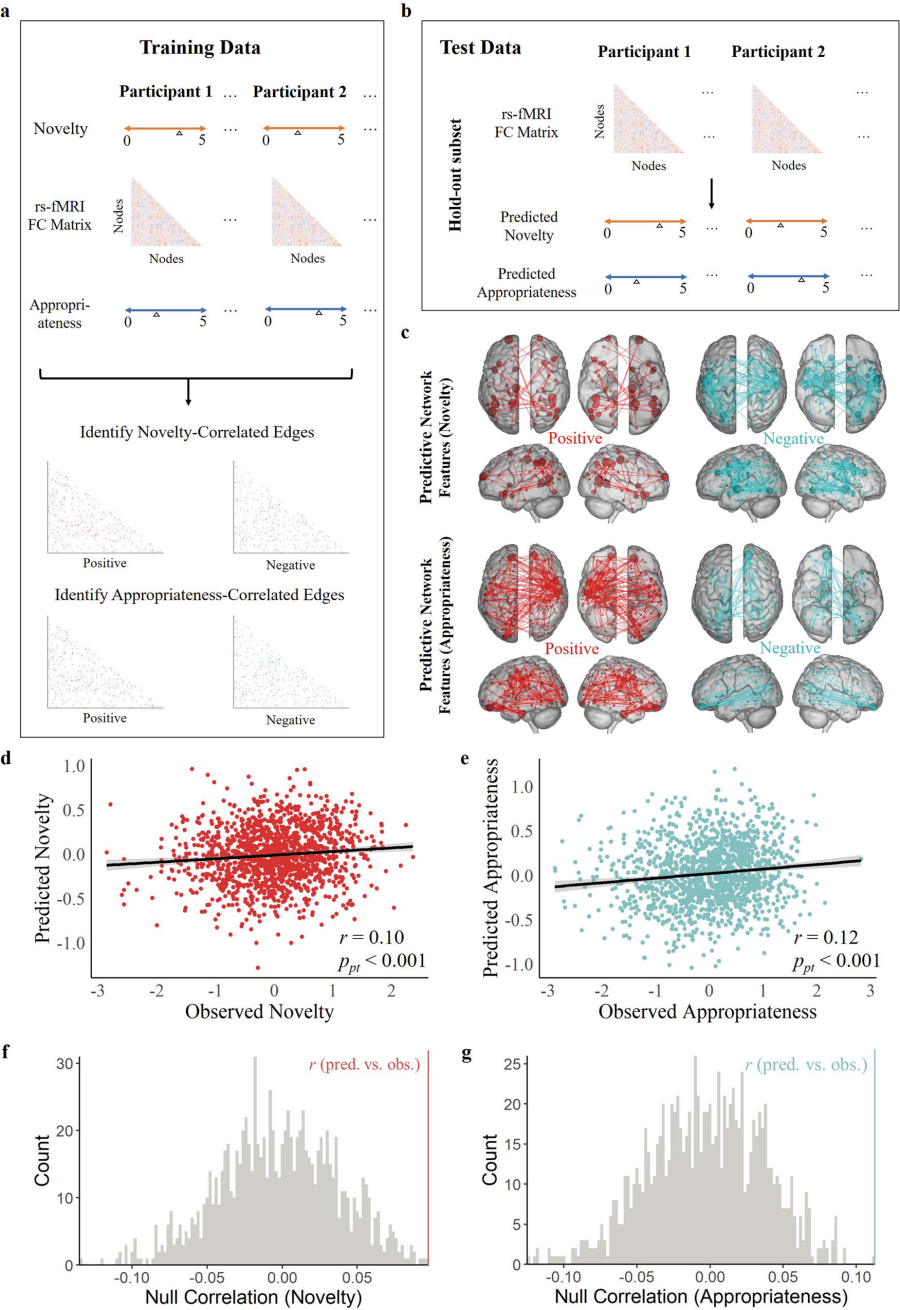
Functional connectivity-based predictive modeling of trial-wise novelty and appropriateness fluctuations. **a** A schematic of the analysis pipeline. Within training data, novelty score (orange arrows), appropriateness score (blue arrows), and functional connectivity matrices (based on a 300-node whole-brain atlas) were extracted for each participant. Red indicates positive functional connectivity of novelty/appropriateness. Blue indicates negative functional connectivity of novelty/appropriateness. Significant edges were identified at a threshold of *p* < 0.01 (uncorrected). **b** Predicted novelty ratings and predicted appropriateness ratings of the held-out subset were calculated based on the training result. **c** Edges strongly contributing positively (red) and negatively (blue) to the predictive model of novelty (top) and the predictive model of appropriateness (bottom). A degree threshold of 1 was applied; i.e., nodes involved in at least one contributing edge are displayed. **d** The correlation between the predicted and observed novelty ratings based on brain connectivity for the significant predictions. Source data are provided as a Supplementary Data file. **e** The corr**e**lation between the predicted and observed appropriateness ratings based on brain connectivity for the significant predictions. Source data are provided as a Supplementary Data file. **f** Correlation value between predicted and observed novelty ratings was compared with a null distribution of *r* values derived from 1000 permutations of shuffled functional connectivity matrix. The gray lines represented the results (*r* values) of 1000 permutations. The red line represented the correlation value between predicted and observed novelty ratings and it (*r* = 0.099) was greater than the *r* values of all 1000 permutations, which showed that the CPM of novelty was significant (*p*_pt_ < 0.001). Source data are provided as a Supplementary Data file. **g** Same as (**f**), except for the predicted and observed appropriateness ratings. The blue line represented the correlation value between predicted and observed appropriateness ratings and it (*r* = 0.121) was greate*r* than the *r* values of all 1000 permutations, which showed that CPM of appropriateness was significant. Source data are provided as a Supplementary Data file.

To examine whether our hypothesis also existed at a neural level, we aimed to identify functional network-based markers of novelty and appropriateness using CPM approach^62^ in rs-fMRI of BBP (*n* = 1455, 981 females, age *M* = 21.1, SD = 0.98). We calculated the functional connectivity matrix based on the whole brain functional map of 300 nodes belonging to 7 functional networks^63^. We adopted relevance vector regression (RVR) to examine the predictive performance of the functional connectome on novelty and appropriateness. Within each cross-validation fold (10-fold cross-validation (10F-CV)), we respectively, identified all node pairs (edges) exhibiting suprathreshold-level (*p* < 0.01) positive and negative correlations with novelty ratings and appropriateness ratings in a training set (90% of participants, Fig. 3a). We put these edges as input features into the RVR model for estimation to get a function that fits the behavior ratings (novelty and appropriateness) to the selected features, respectively. Next, the estimated RVR model was used on the test set (the remaining 10% of participants) to obtain predicted behavioral ratings (novelty and appropriateness) (Fig. 3b). After all folds were completed, we obtained the predicted ratings for each participant. Because each random division results in different testing sets and training sets, we repeated the above prediction pipeline 150 times to generate 150 predicted ratings for each participant and further averaged these predicted ratings to obtain robust estimates. We finally correlated the averaged predicted ratings with the observed rating and then compared the correlation value with a null distribution (see “Methods” for detailed descriptions).

The permutation results showed that CPM of novelty (*r* = 0.099, *p*_pt_ < 0.001) and CPM of appropriateness (*r* = 0.121, *p*_pt_ < 0.001) were both effective (Fig. 3d–g). This provided evidence that our functional network model predicted novelty and appropriateness within individuals at the within-dataset level (i.e., establishing internal validation) and with an overall effect size that was on par with that typically found for functional connectivity-based prediction of self-report outcomes^64,65^. The edges contributing to the models included 296 and 430 edges positively and negatively associated with novelty and appropriateness, respectively (hereafter referred to as “novelty–CPM” and “appropriateness–CPM” masks). These edges of novelty–CPM and appropriateness–CPM were both distributed widely throughout the brain, with novelty–CPM high-degree nodes (i.e., nodes involved in multiple contributing edges) situated in temporal, prefrontal, parietal, and occipital cortices and with appropriateness–CPM high-degree nodes situated in prefrontal, temporal, and parietal cortices (Fig. 3c).

### Validity and specificity of the novelty–CPM and appropriateness–CPM

We performed a series of confirmatory analyses to assess the validity and specificity of the novelty–CPM and appropriateness–CPM. In order to avoid the arbitrariness of a single threshold, we additionally applied two common thresholds (uncorrected *p* value: 0.05 and 0.005) for feature selection. We found that both novelty–CPM (*r*_0.05_ = 0.080, *p*_pt_ = 0.011; *r*_0.005_ = 0.094, *p*_pt_ = 0.005) and appropriateness–CPM (*r*_0.05_ = 0.125, *p*_pt_ < 0.001; *r*_*0.005*_ = 0.109, *p*_pt_ = 0.001) remain significant under these two thresholds. Moreover, although fMRI preprocessing steps can reduce the impact of noise on functional connectivity, frame-wise head motion can still affect the relationship between functional connectivity and behavior^66,67^. Therefore, we re-conducted the CPM model under three thresholds (uncorrected *p* value: 0.05, 0.01, and 0.005) while controlling for mean framewise head motion and common demographic variables (age, gender, and handedness). We also found that both novelty–CPM (*r*_0.05_ = 0.075, *p*_pt_ = 0.013; *r*_0.01_ = 0.097, *p*_pt_ = 0.004; *r*_0.005_ = 0.089, *p*_pt_ = 0.007) and appropriateness–CPM (*r*_0.05_ = 0.127, *p*_pt_ < 0.001; *r*_0.01_ = 0.119, *p*_pt_ = < 0.001; *r*_0.005_ = 0.114, *p*_pt_ < 0.001) remain significant when using partial correlations for feature selection, controlling for mean framewise head motion, age, gender and handedness.

**Fig. 4:**
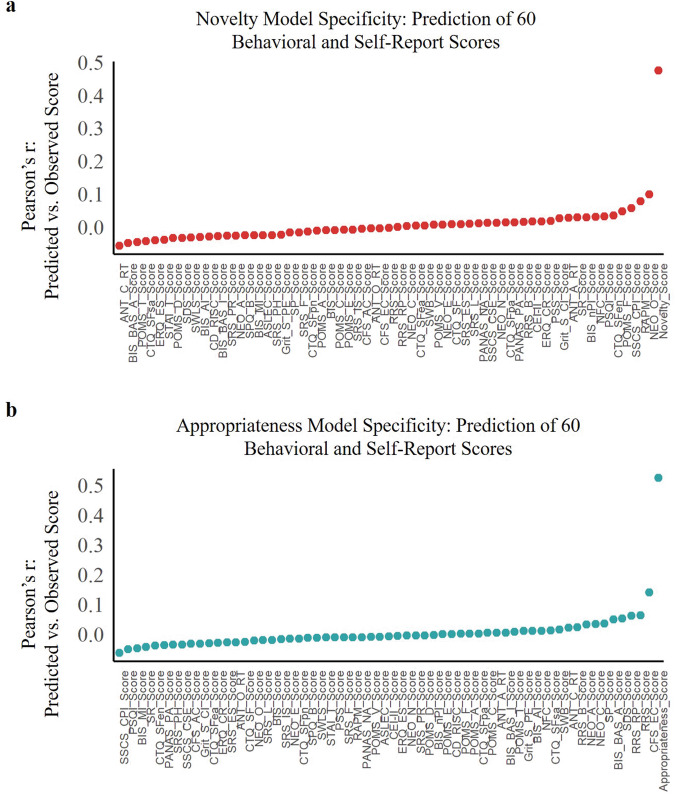
The specific functional neuroanatomical basis for novelty and appropriateness derived from rs-fMRI (BBP dataset). **a** Correlations between novelty–CPM prediction and 60 behavioral and self-report outcomes in the BBP dataset. Among all outcomes, novelty (denoted as “Novelty_Score”) showed the highest correlation with model prediction. **b** Same as (**a**), except for the appropriateness–CPM and appropriateness (denoted as “Appropriateness_Score”). See Supplementary Methods for a phenotype legend of labels shown. CPM connectome-based predictive model. Source data are provided as a Supplementary Data file.

To illustrate the specificity of functional connectivity patterns that predicted the observed novelty score and observed appropriateness score, we conducted the Pearson’s correlation between novelty/appropriateness–CPM predictions from rs-fMRI and a comprehensive battery of 60 distinct behavioral and self-report individual outcomes that were obtained in addition to novelty and appropriateness ratings (see Supplementary Methods). Among this entire set of individual outcomes, the novelty prediction showed the strongest correlation with observed novelty ratings (Fig. 4a), and the appropriateness prediction showed the strongest correlation with observed appropriateness ratings (Fig. 4b). However, inconsistencies in sample size caused by data matching between behavioral outcomes and functional connectivity matrixes from rs-fMRI may bring comparison errors, we therefore repeated the above process using effect size (*η*^2^), which is not affected by sample size^68^. We still found that novelty–CPM has the highest effect size on observed novelty ratings (see Table S2) and appropriateness–CPM has the highest effect size on observed appropriateness ratings (see Table S3).

### Functional neuroanatomical basis of the novelty and appropriateness networks

**Fig. 5:**
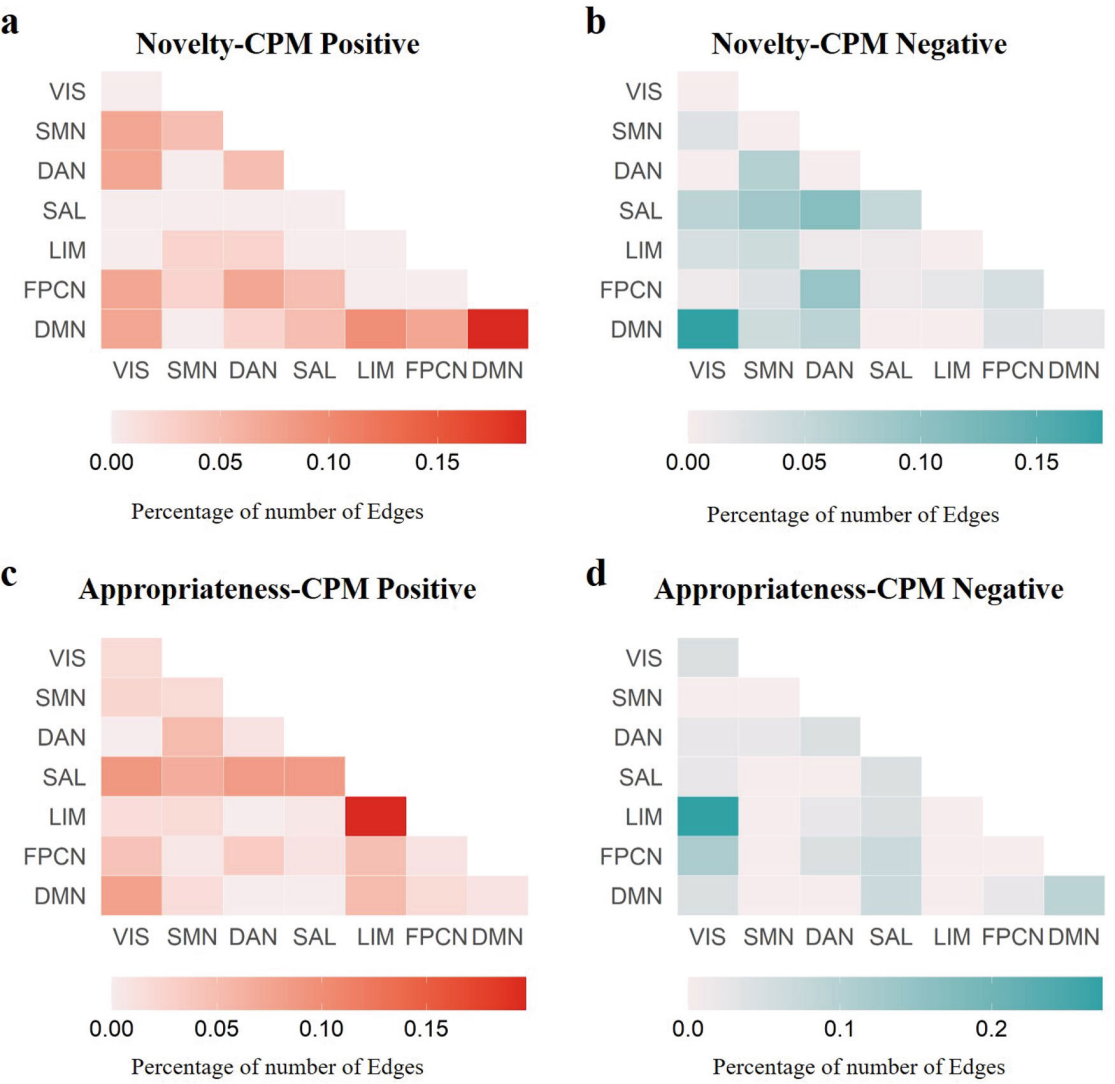
Functional neuroanatomical basis of the novelty–CPM and appropriateness–CPM network. **a** The percentage of the number of edges, among those within the novelty–CPM positive mask, assigned to each within-network or between-network pair based on the Schaefer300 and Yeo–Krienen 7-network atlases. **b** Same as (**a**), except for the novelty–CPM negative mask. **c** Same as (**a**), except for the appropriateness–CPM positive mask. **d** Same as (**c**), except for the appropriateness–CPM negative mask. DAN dorsal attention network, DMN default mode network, FCPN frontoparietal control network, LIM limbic network, SAL salience network, SMN sensorimotor network, VIS visual network, CPM connectome-based predictive model. Source data are provided as a Supplementary Data file.

To better explain the functional neuroanatomical basis of patterns contributing to the novelty–CPM and appropriateness–CPM, we examined the relationships with the functional networks previously associated with novelty and appropriateness. According to the Schaefer300 atlas, with each node assigned to 1 of 7 standard Yeo–Krienen networks^69^, we quantified the number of novelty–CPM and appropriateness–CPM mask edges belonging to each intra- or inter-network pair. The highest number of edges positively correlated with novelty (Fig. 5a) derived from within-network and between-network connections of the DMN. The top three network pairs contributing to positive edges were DMN–DMN, DMN–LIM, and DMN–FPCN. The DMN–DMN connections contributed the most positive edges (19.05%). The top three network pairs contributing to edges negatively correlated with novelty were DMN–visual network (VIS), dorsal attention network (DAN)–SAL, and DAN–FPCN (Fig. 5b). The DMN–VIS connections contributed the most negative edges (17.80%).

The highest number of edges positively correlated with appropriateness (Fig. 5c), derived from SAL within-network and between-network connections. The top three network pairs contributing to edges positively correlated with appropriateness were limbic network (LIM) within-network, SAL–VIS, and LIM–SAL. The LIM–LIM connections contributed the most positive edges (19.72%). The highest number of edges negatively correlated with appropriateness (Fig. 5d), derived from VIS within-network and between-network connections. The top three network pairs contributing to negative edges were VIS–LIM, VIS–FPCN, and DMN–DMN. The VIS–LIM connections contributed the most positive edges (27.27%). These results collectively suggest that novelty and appropriateness were supported by different functional brain network-based markers.

To further illustrate the robustness of novelty–CPM and appropriateness–CPM, we compared the results of the 6 models at the network level. As shown in Figs. 5 and S1, they are visually similar, especially in the top one network pairs: DMN–DMN for positive network pairs of novelty–CPM; DMN–VIS for negative network pairs of novelty–CPM; LIM–LIM for positive network pairs of appropriateness–CPM; VIS–LIM for negative network pairs of appropriateness–CPM. We next calculated the average correlation coefficients of the six models in the four network pairs. The results showed that the positive (*r* = 0.821 ± 0.106) and negative (*r* = 0.862 ± 0.062) network pairs of novelty–CPM, and the positive (*r* = 0.789 ± 0.128) and negative (*r* = 0.758 ± 0.161) network pairs of appropriateness–CPM between the six models had strong correlations. This analysis demonstrated that both the novelty–CPM and the appropriateness–CPM exhibit strong robustness.

### External validation #1: applying the connectome predictive models to predict novelty and appropriateness in an independent rs-fMRI dataset

**Fig. 6:**
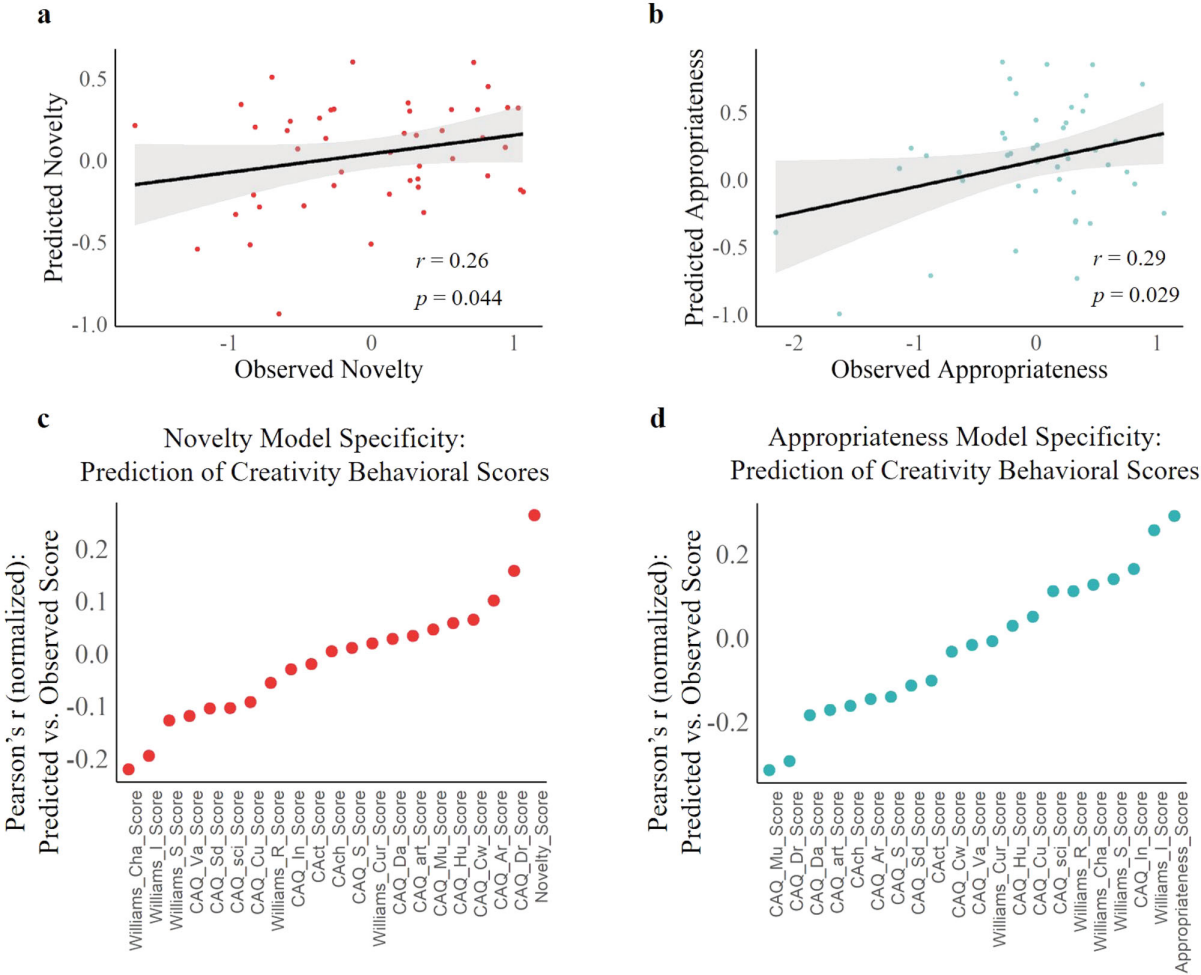
The external validation and specificity of novelty–CPM and appropriateness–CPM on the EV1 dataset. **a** The plot shows the correlation between the predicted and observed novelty ratings based on resting-state brain connectivity from the EV1 dataset. **b** Same as (**a**), except for the predicted and observed appropriateness ratings. **c** Correlations between novelty–CPM prediction and creativity behavioral scores in the EV1 dataset. Among all outcomes, novelty (denoted as “Novelty_Score”) showed the highest correlation with model prediction. **d** Same as (**c**), except for the appropriateness–CPM and appropriateness (denoted as “Appropriateness_Score”). See Supplementary Methods for a phenotype legend of labels shown. CPM connectome-based predictive model. Source data are provided as a Supplementary Data file.

Having established the functional neuroanatomical basis of novelty–CPM and appropriateness–CPM based on rs-fMRI, we next explored whether the predictive models can be generalized to the prediction of novelty and appropriateness from the resting-state functional connectivity in another independent dataset (*n* = 46, 37 females, age *M* = 21.7, SD = 1.92) (hereafter referred to as “EV1 dataset”). To this end, we used the positive- and negative- models from the resting-state functional connectivity data from the BBP dataset to build the predictive model for novelty and appropriateness (using the same method as the BBP dataset prediction described above). We next applied novelty–CPM and appropriateness–CPM to the functional connectivity of each participant from the new rs-fMRI dataset to compute the predicted novelty/appropriateness ratings, respectively. The model prediction was assessed by Pearson’s partial correlation between the predicted value from the model and the observed values (controlling for mean framewise head motion, age, and gender). We found a significant positive correlation between novelty ratings and novelty–CPM prediction from resting-state functional connectivity (*r* = 0.26, *p* = 0.044, one-tailed, Fig. 6a). The appropriateness–CPM prediction of resting-state connectivity was positively correlated with appropriateness ratings (*r* = 0.29, *p* = 0.029, one-tailed, Fig. 6b).

We have illustrated the specificity of novelty–CPM and appropriateness–CPM using several distinct behavioral and self-report individual outcomes in the BBP dataset. However, these behaviors do not directly measure creativity. Therefore, in this dataset, we used behavioral outcomes measuring real-life creativity and creative tendencies including the creative achievement questionnaire^70^, inventory of creative activities and achievements^71^, and Williams’ creativity assessment packet^72^ to further verify the specificity of novelty–CPM and appropriateness–CPM for divergent thinking. Among this entire set of individual outcomes, novelty prediction showed the strongest correlation with the observed novelty score (Fig. 6c), and appropriateness prediction showed the strongest correlation with the observed appropriateness score (Fig. 6d). Because the data involved here had consistent sample sizes, we did not repeat this part of the analysis further using effect size.

### External validation #2: applying the connectome predictive models to predict novelty and appropriateness in an independent task-fMRI dataset

We have so far established a functional network model that carries predictive information about novelty and appropriateness, measured during rs-fMRI. In the next analysis, we focused on whether the novelty–CPM and appropriateness–CPM are sensitive to task performance of novelty and appropriateness, respectively, based on task-fMRI. We used task-fMRI of AUT from another independent dataset (*n* = 31, 24 females, age *M* = 21.8, SD = 1.88) to examine the generalizability of novelty–CPM and appropriateness–CPM. In each trial of this paradigm (Fig. S2), an object was presented on screen (e.g., hair, see Table S4 for details of the objects presented in each trial). In the novelty use (NU) condition (31 s per trial), participants were asked to think of as many novel and appropriate uses as they could for each object. In the general characteristic (GC) condition (21 s per trial), participants were asked to think of as many GCs as they could for each object (see “Methods” for details of the stimulus presentation and procedure). Participants responded verbally using a microphone. In the analysis of task-fMRI data, only the NU condition was considered.

**Fig. 7:**
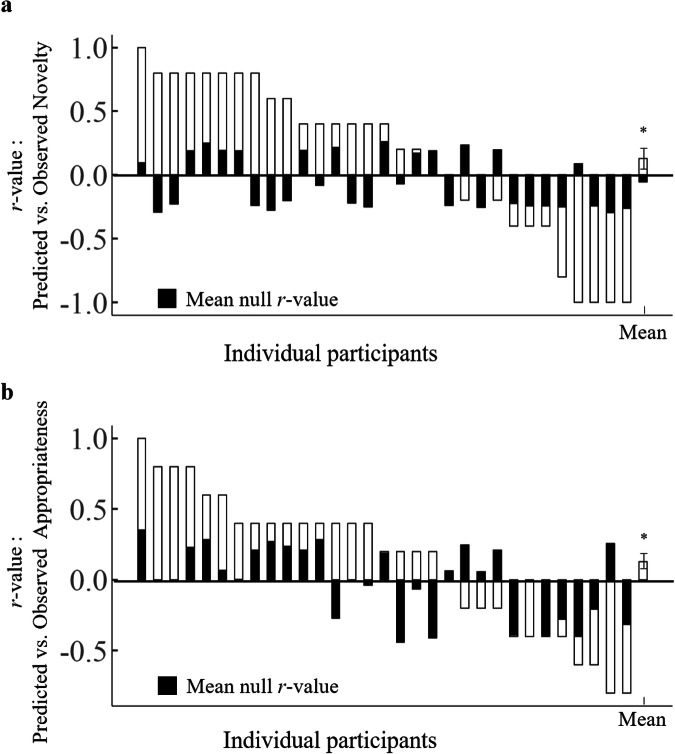
The external validation of novelty–CPM and appropriateness–CPM on task-fMRI. **a** Predicted vs observed novelty ratings Spearman correlations, and mean null correlations, within each held-out participant. The last bar (far right) indicates the mean across individuals (i.e., mean across white bars; *n* = 31). At the group level, predicted vs observed correlations were significantly greater than mean null correlations (*p* = 0.047, one-tailed, Wilcoxon signed-rank test). **b** Same as (**a**), except for the CPM prediction of appropriateness and appropriateness ratings. CPM connectome-based predictive model. *p < 0.05. Error bars indicate the standard error of the mean. Source data are provided as a Supplementary Data file.

We applied novelty–CPM and appropriateness–CPM to the functional connectivity of each block for each participant to compute the predicted novelty/appropriateness ratings for each block, respectively (refer to previous research analysis^64^ and see “Methods” for details). We next calculated the Spearman rank correlation between predicted novelty/appropriateness and observed novelty/appropriateness, respectively, and then compared the correlation value with a null distribution. Correlations between novelty–CPM prediction (based on the rs-fMRI of BBP dataset) and observed novelty (from task-fMRI) were significantly greater than mean null values (mean *r* = 0.13, *p*_pt_ = 0.047, one-tailed, Wilcoxon signed-rank test) (Fig. 7a). Moreover, appropriateness–CPM had also been successfully verified (mean *r* = 0.13, *p*_pt_ = 0.043, one-tailed, Wilcoxon signed-rank test) (Fig. 7b).

### Mediation analysis

In the previous analyses, we found that the generation of more novel ideas relied more on associative abilities, while the generation of more appropriate ideas relied more on executive functions. We also found that novelty–CPM and appropriateness–CPM can predict novelty and appropriateness, respectively. In a final step, we analyzed whether the relationship between novelty and associative abilities is mediated by the novelty–CPM; and whether the relationship between appropriateness and executive functions is mediated by the appropriateness–CPM. This analysis used the data from the BBP dataset.

**Fig. 8:**
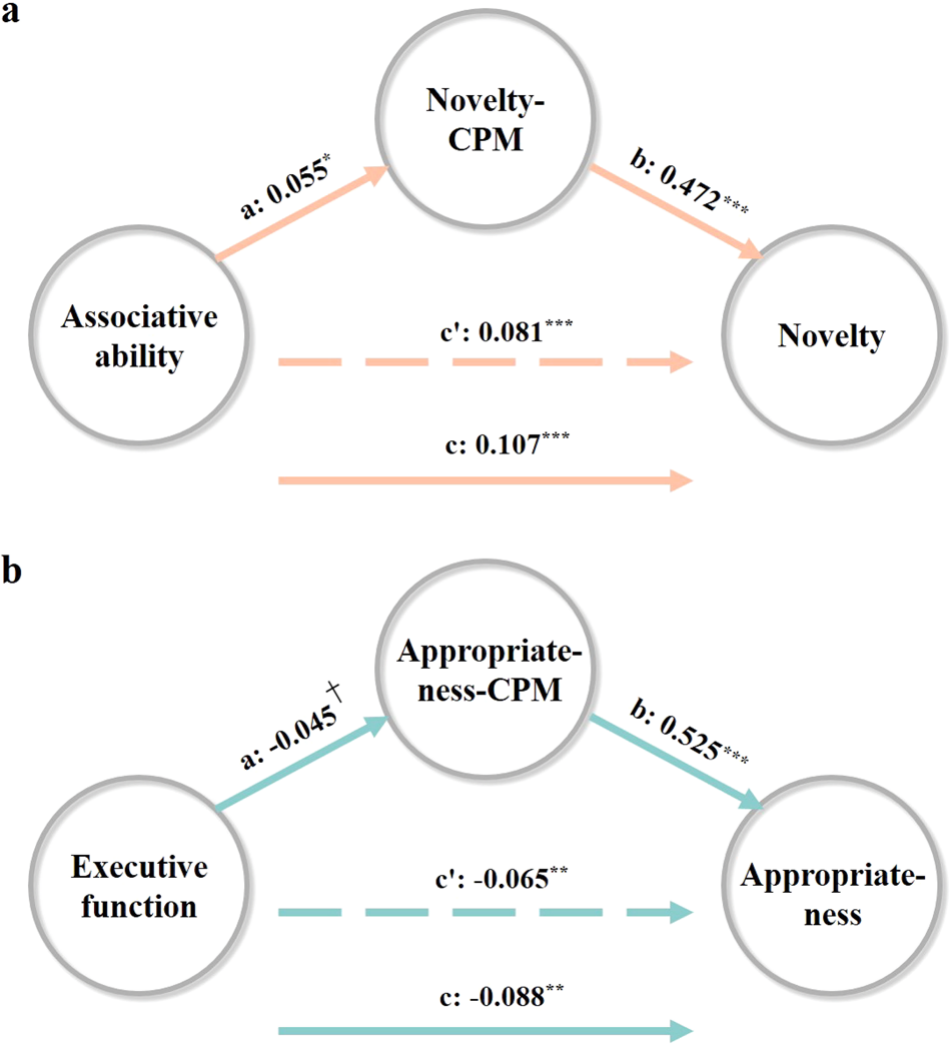
Mediation analyses. Results of the mediation models are presented in path diagrams. Each diagram indicates the beta weights of the regression coefficients. The total effect is indicated by path c, the direct effect by path c′, and the indirect effect is given by the product of path a and path b. **a** The mediating role of novelty–CPM on the relationship between the associative abilities and novelty ratings. **b** The mediating role of appropriateness–CPM on the relationship between the executive functions and appropriateness ratings. ^†^*p* = 0.098, *p < 0.05, **p < 0.01, and ***p < 0.001. Source data are provided as a Supplementary Data file.

We explored the mediating role of novelty–CPM on the relationship between the associative abilities and novelty ratings (Fig. 8a). As shown in the previous analyses, the regression coefficient between novelty ratings and novelty–CPM was statistically significant (*β* = 0.472, *p* < 0.001), as was the regression coefficient between associative abilities and novelty–CPM (*β* = 0.055, *p* < 0.05). The total effect and the direct effect were statistically significant (*β* = 0.107, *p* < 0.001; *β* = 0.081, *p* < 0.001). We tested the significance of the indirect effect using a bootstrapping method. The bootstrapped indirect effect was (0.055) × (0.472) = 0.026, and the 95% confidence interval ranged from 0.001 to 0.065. Thus, the indirect effect was statistically significant. Hence, novelty–CPM mediated the relationship between associative abilities and novelty ratings.

We next explored the mediating role of appropriateness–CPM on the relationship between the executive functions and appropriateness ratings (Fig. 8b). As shown in the previous analyses, the regression coefficient between appropriateness ratings and appropriateness–CPM was statistically significant (*β* = 0.525, *p* < 0.001), and the regression coefficient between executive functions and appropriateness–CPM was marginally significant (*β* = −0.045, *p* = 0.098). The total effect and the direct effect were statistically significant (*β* = −0.088, *p* < 0.01; *β* = −0.065, *p* < 0.01). We tested the significance of the indirect effect using a bootstrapping method. The bootstrapped indirect effect was (−0.045) × (0.525) = −0.024. Although the mediation model is not significant at the 95% confidence interval, it is significant at the 90% confidence interval (ranging from −0.078 to −0.002).

In addition, we also found that appropriateness was predicted by associative abilities (Fig. 2b orange). Therefore, we finally explored the mediating role of appropriateness–CPM on the relationship between associative abilities and appropriateness ratings (Fig. S3). The regression coefficient between appropriateness ratings and appropriateness–CPM was statistically significant (*β* = 0.525, *p* < 0.001), and the regression coefficient between associative abilities and appropriateness–CPM was statistically significant (*β* = −0.061, *p* < 0.05). The total effect and the direct effect were statistically significant (*β* = −0.078, *p* < 0.01; *β* = −0.046, *p* < 0.05). We tested the significance of the indirect effect using a bootstrapping method. The bootstrapped indirect effect was (−0.061) × (0.525) = −0.032, and the 95% confidence interval ranged from −0.077 to −0.008.

## Discussion

The present study provides evidence for the separability of novelty and appropriateness of creative idea generation at both the behavioral and neural levels. Previous work has associated localized differences in brain activity with the generation of novel and appropriate ideas by manipulating conditions of idea generation^56–58^. The present study takes a global and predictive approach, developing data-driven models of whole-brain functional connectivity that distinguished and predicted novelty and appropriateness across brains and between resting state and task-based fMRI. Additionally, a separate behavioral component of the present study supported a theory-driven framework for distinguishing divergence and convergence based on associative vs executive processes. Together, the present data indicate that the novelty and appropriateness of creative idea generation are discernably independent, and identify distinct cognitive bases and neural network signatures for these two primary components of creativity.

Our results found that novelty was only predicted by associative abilities, but not executive functions (Fig. 2a). This finding aligns with the associative theory of creativity, which holds that novelty of creative ideas**/**products represents an ability to produce distant associations^18^. Although novelty is an important aspect of creativity, solely pursuing novelty in creative activities is not practical or realistic. Creative outcomes should be balanced in terms of novelty and appropriateness. For a specific creative activity, individuals first must possess certain knowledge and experience related to the current creative activity before they can generate an appropriate product or idea^10^. Furthermore, an individual’s knowledge and experiences are complex and require top–down executive processes to extract and process the knowledge and experience related to the current creative activity. This may explain why the generation of more appropriate ideas relies more on executive functions (Fig. 2b, blue line). Specifically, cognitive flexibility is responsible for screening as much information as possible; inhibitory control is responsible for excluding irrelevant information; and working memory is responsible for providing storage space for the necessary information processing activities. Moreover, our results revealed that associative abilities had a negative impact on appropriateness (Fig. 2b, orange line). This implies that while associative abilities enhance the novelty of generated ideas, it also hinder their appropriateness. This finding helps to explain the negative relationship between novelty and appropriateness to a certain extent^73–75^.

Using CPM, we uncovered distinct whole-brain networks associated with novelty and appropriateness. Network edges identified for both novelty–CPM and appropriateness–CPM were widely distributed throughout the brain, with novelty–CPM high-degree nodes (i.e., nodes involved in multiple contributing edges) situated in temporal, prefrontal, parietal, and occipital cortices, and with appropriateness–CPM high-degree nodes situated in prefrontal, temporal, and parietal cortices (Fig. 3c). Our findings show that the distributed nature of interactions within and between different brain networks was critical for the generalizability of novelty–CPM predictions and appropriateness–CPM predictions. Notably, the connectomic signatures of novelty and appropriateness exhibited both distinct and overlapping components. Specifically, DMN within-network (positive support) and DMN–VIS (negative support) distinctly related with novelty, LIM within-network (positive support) and LIM-VIS (negative support) connections distinctly related to appropriateness (Fig. 5). This illustrates that the generation of novel ideas is more supported by DMN, while the generation of appropriate ideas is more supported by LIM. Whether generating novel or appropriate ideas, VIS was an overlapping component, and showed decreased synchronization with other networks (DMN and LIM).

The distinct functional connectivity of novelty is remarkably similar to patterns of functional connectivity reported in prior research of creative cognition^8,13^. Specifically, we found that the greatest number of significantly correlated interactions occurred within the hub of one large-scale brain network: DMN (Fig. 5a). Our findings are consistent with a large body of evidence that DMN activity during free association correlates with behavioral measures of divergent thinking, suggesting that the DMN may play a role in free association during creative thinking^76,77^. Notably, neuroimaging studies have also reported that artistic performance, music composition, and real-life creative achievement are also associated with functional coupling within regions in this network^11,78–81^, which may reflect a domain-general mechanism of creative information processing.

For the distinct neuroanatomy of appropriateness, the distributed nature of interactions within LIM supported the generation of appropriate ideas (Fig. 5c). These findings extend previous findings on the neural basis of appropriateness^56–58^. LIM contains two brain areas, the temporal pole (TP) and OFC^69^. Multiple functions have been ascribed to the OFC including mediating context-specific responding^82^, encoding contingencies in a flexible manner, encoding value, encoding inferred value, inhibiting responses, learning changes in contingency, emotional appraisal^83^, altering behavior through somatic markers, driving social behavior, and representing state spaces^84^. However, specific functions have been ascribed to subregions of the OFC. The lateral OFC has been proposed to reflect potential choice value, enabling fictive (counterfactual) prediction errors to potentially mediate switching choices during reversal, extinction, and devaluation^85^. On the other hand, TP is a part of the temporal lobe, which is mainly responsible for processing speech information and also has the function of assigning meaning to sound information, especially the left TP^86^. This may indicate that during the process of generation of creative ideas, individuals evaluate the value of the ideas generated to ensure their appropriateness.

Previous research has demonstrated that the executive control network plays an important role in creativity^13,87^, but our results suggested that FPCN is less weighted in both novelty–CPM and appropriateness–CPM (Fig. 5c). It is particularly surprising that the FPCN does not feature prominently in appropriateness neuroanatomy (appropriateness–CPM), as the FPCN plays a central role in executive control functions^88^ that are relied upon more to generate more appropriate ideas. This may be a result of the separate investigation of novelty and appropriateness in the present study, whereas the FPCN may be required for the integration of novelty and/or appropriateness. Psychological theory proposes that creativity involves a dual process, including a generative process that connects distant concepts and an evaluative process that can assess their novelty and appropriateness^6^. An empirical study designed an fMRI paradigm with high ecological validity, requiring participants to design illustrations for book covers, which distinguished the creative generation and evaluation processes at the neural level^78^. The study found that FPCN was significantly activated and showed positive functional connectivity with DMN for both generation and evaluation processes. Therefore, while it is beneficial to gain a deeper understanding of creativity by examining novelty and appropriateness separately, it is also important to consider research findings that investigate creativity as a unified construct. By integrating both perspectives, we can obtain a more comprehensive understanding of the creative process and its neural underpinnings.

Both in novelty–CPM and appropriateness–CPM negative mask, our results found a dominant role for the VIS. One explanation for these results is that highly creative people are more likely to engage in internally directed cognition in the absence of external tasks^89,90^. In other words, individuals who are able to generate more novel and appropriate ideas are better able to control themselves from being disturbed by external stimuli, which is manifested in the reduction of synchrony between VIS and other brain network activities.

To further illustrate the specificity of novelty–CPM and appropriateness–CPM for novelty and appropriateness, respectively, our results showed that the prediction effects of novelty–CPM and appropriateness–CPM on other creativity scores were not significant (Fig. 6). Divergent thinking is considered a creative potential and does not guarantee actual creative performance (e.g., creative achievement)^91^. Although a large amount of evidence shows that divergent thinking can effectively predict creative performance^35,92–95^, the difference between these constructs cannot be ignored. One study found that divergent thinking and creative performance were predicted by different factors (e.g., general intelligence and domain knowledge)^96^. Therefore, we believe that this difference is also reflected in functional brain activity, as demonstrated by our results (Fig. 6). However, it is important to note that our results provide indirect evidence, and further research is needed to explore this issue more directly and comprehensively in the future.

Finally, we found that the relationship between novelty and associative abilities is mediated by novelty–CPM (Fig. 8a); the relationship between appropriateness and executive functions is mediated by appropriateness–CPM (Fig. 8b); the relationship between appropriateness and associative abilities is mediated by appropriateness–CPM (Fig. S3). These results link the dissociation of novelty and appropriateness at behavioral and neural levels. On the one hand, associative abilities and executive control, as basic cognitive abilities, have been shown to be closely linked to creativity^27,97^. In addition, creativity can be effectively improved through training on associative abilities or executive functions^98–101^. Our mediation results provided a possible neural basis for this creative intervention-enhanced path. In line with, previous research demonstrating that brain network activity and connectivity can be affected through cognitive training^102–104^, it is conceivable that long-term training of associative abilities and executive functions could alter individual brain functional connections, resulting in the improvement of creative abilities.

The present study contains several key limitations. First, we analyzed the functional neuroanatomical basis of novelty–CPM and appropriateness–CPM networks using the number of edges (Fig. 5). Although this can reflect the weight of brain network interactions on which novelty and appropriateness depend, only considering the number of edges may not reveal the full picture of the neural basis of novelty and appropriateness. Therefore, more research is needed to replicate our findings, using alternative metrics such as edge strength to assess novelty and appropriateness. Second, only two AUT items were used to measure individual divergent thinking in our research. As suggested by Beaty et al., using only two AUT items may not capture the domain-general construct of divergent thinking^105^. Therefore, our findings require further validation using a number of divergent thinking tasks. Finally, we employed CPM to identify distinct whole-brain networks associated with novelty and appropriateness, respectively. Perhaps due to the limitations of research methods, we did not find the functional connectivity shared by novelty and appropriateness. Future research aimed at understanding the neural basis shared by novelty and appropriateness may lead to a more comprehensive understanding of creativity.

Taken together, our findings distinguish novelty and appropriateness of idea generation at both the cognitive and neural levels. Future research, should examine the interaction of novelty and appropriateness during creative idea generation.

## Methods

### Participants

Behavioral and neuroimaging data were obtained at the Southwest University. All participants provided written informed consent and received payment for their time and task participation, and the research protocol was approved by the ethics committee of the review committee of the Brain Imaging Center of Southwest University.

Three independent datasets were included in this study. The first dataset comprised individuals in the BBP, from which behavioral and rs-fMRI data were obtained for the present study. In this dataset, we first analyzed the behavior data to test whether novelty draws more on associative abilities and whether appropriateness draws more on executive functions. Therefore, only participants who performed the alternate uses task (AUT), FFT and three executive functions tasks (b-back task, stop signal task, and number–letter category switching task) were eligible to participate (*n* = 1535). Participants with outliers (defined as *z*-scores of all values of behavioral tests exceeding 3 or −3) were excluded from this analysis. This final analysis included 1509 participants (mean age 21.1 ± 0.97 years; 1014 females). Next, we identified functional network-based markers of novelty and appropriateness using a connectome-based predictive model (CPM) approach in rs-fMRI of BBP. Therefore, only participants who underwent resting-state scans and performed the AUT were eligible to participate (*n* = 1547). Participants with mean overall frame-wise displacement (FD) of >0.20 mm (based on the power method^106^) during rs-fMRI were excluded from this analysis. This final analysis included 1455 participants (mean age 21.1 ± 0.98 years; 981 females).

The second dataset was used to verify the generality of the predictive models derived from the BBP dataset. This dataset is called the EV1 dataset in this study. This dataset included a total of 46 participants (mean age 21.7 ± 1.92 years; 37 females).

The third dataset, a task-fMRI dataset, was used to verify whether the predictive models derived from the BBP dataset can be generalized to data with different modalities. This dataset is called the EV2 dataset in this study. This dataset included a total of 55 participants and 24 participants were excluded due to not meeting the analysis conditions (see “Methods” “task-fMRI of AUT” for details). This final analysis included 31 participants (mean age 21.8 ± 1.88 years; 24 females).

### Assessment of divergent thinking

#### AUT

In the BBP implementation of the AUT, participants were asked to generate as many novel and appropriate uses as they could think of for a “Brick” and a “Can” within a 3-min time frame^95^. Each object (e.g., “Brick”) was presented on the screen for 3 min. When participants came up with an idea, they were instructed to press the “1” button and then to type their idea. This was repeated until the end of the 3-min period. The participants were presented with the following instructions: “Please write down any interesting and unusual uses you can imagine for these. You can try to come up with uses that others haven’t thought of, and the more ideas you have, the better, and the more novel they are, the better.” E-prime 2.0 was used to display this task.

Based on the methodologies used in previous studies^95,107^, four previously trained raters evaluated the AUT responses. All responses of the two items were assessed on four aspects of creativity: fluency, which refers to the number of responses for each object; flexibility, which measures the number of distinct categories into which the responses can be classified; novelty, which measures the originality of each response, using a 5-point scale where a “1” is very unoriginal and a “5” is very original; and appropriateness, which assesses how suitable each response is for the particular object (i.e., whether the suggested use is meaningful or nonsensical) using a 5-point scale where a “1” is very unsuitable and a “5” is very suitable. An analysis of the rater agreement showed good inter-rater reliability, ranging from 0.728 to 0.984.

In the EV1 dataset, the administration of the AUT was consistent with the BBP dataset, with the only exception being that participants did not use E-prime for testing. An analysis of the rater agreement showed good inter-rater reliability, ranging from 0.736 to 0.919.

In our study, only novelty and appropriateness ratings were included in the analysis. We averaged the scores of the four raters for both the novelty and appropriateness of the two objects (“Can” and “Brick”). We then averaged the standardized scores of novelty and appropriateness of the two items.

#### Product improvement task (PIT)

In the EV1, PIT requires an individual to create ideas to make a toy elephant more fun, enjoyable, and appealing in a 10-min time period^108^. The method of evaluation for this task is exactly the same as the AUT. An analysis of the rater agreement showed good inter-rater reliability, ranging from 0.683 to 0.791. In our study, only novelty and appropriateness ratings were included in the analysis. We averaged the ratings of the four raters for both the novelty and appropriateness of the PIT.

In the EV1 dataset, the average ratings of AUT and PIT (novelty and appropriateness) were used for external validation of novelty–CPM and appropriateness–CPM as an assessment of divergent thinking.

### Assessment of associative abilities

#### FFT

The FFT was also used to measure associative abilities. In the FFT, a cue word was presented, and participants were then asked to write down the first thought they could think of related to the cue word. Each subsequent thought should be based on the previous thought (e.g., cake: “birthday, candle, lighting, …”). There were two items (“cake” and “snowflake”) for this task and participants were instructed to generate as many words as possible in 1 min for each item.

We used semantic distance^9,25^ to evaluate associative abilities. To calculate the semantic distance of FFT, our approach used word2vec^22^, one of a number of natural language processing technologies in which patterns of word co-occurrence are used to construct a high-dimensional semantic space (300 dimensions in this study). This method utilizes a large number of natural language corpora, which are divided into many discrete contexts. The corpora are used to generate a co-occurrence matrix to record the frequency of each word in each context. Then a data reduction technique is applied to the matrix so that each word is represented as a high-dimensional vector. Words used in similar contexts (and therefore assumed to have related meanings) are assigned similar vectors. The word similarity obtained in this way is a powerful predictor of human judgment on semantic relevance and human performance in a series of tasks^109,110^. We used the following equation to calculate the semantic distance of FFT for every participant after transforming words into 300-dimensional numerical vectors; where *D* is the semantic distance between thoughts and n is the total number of thoughts within a stream.

$$\left({{\sum}_{i=2}^{n}}\frac{{\sum }_{j=1}^{i-1}{D}_{i,j}}{i-1}\right)/({{{{{\rm{n}}}}}}-1)$$

Finally, we averaged the standardized semantic distance of the two items. The standardized semantic distance of FFT represented the associative abilities; the higher the mean score, the better the associative abilities.

### Assessment of executive functions

#### n-Back task

This task was used to measure working memory in executive functioning. We used only one condition (2-back) for the letter n-back task. Participants were asked to detect whether the current item had flashed two items earlier in the sequence. Participants were instructed to press “F” when the current item matched two items earlier and to otherwise press “J”. Participants completed 90 trials. Each trial lasted 3000 ms and the letter for each trial was presented for 750 ms. The psychophysics toolbox (http://psychtoolbox.org/) for MATLAB was used to display the stimuli.

Mean RT was calculated after excluding the trials with no response and incorrect response. ACC was also calculated after excluding the trials with no response.

#### Stop signal task

The stop signal task was used to measure inhibitory control in executive functioning. This task consisted of four blocks, each of which contained 16 stop trials and 48 go trials. Stop trials and go trials were randomly presented. Each trial began with a central fixation cross for 500 ms, and then a black arrow pointing left or right in a black circle was displayed on the screen. The arrow lasted for 1000 ms for each trial. For the go trials, participants were asked to respond as quickly and accurately as possible by pressing the “F” (for left arrow) or “J” (for right arrow) button within 1000 ms. For the stop trials, the black circle turned red after a period of time. This period of time is the stop-signal delay (SSD), which was dynamically adjusted according to the participant’s response in this task. Participants were asked to withhold the response they already initiated. The initial SSD value is divided into four ladders: Ladder 1 = 140 ms, Ladder 2 = 180 ms, Ladder 3 = 220 ms, and Ladder 4 = 260 ms. The SSD of each participant’s first stop trial was Ladder 3 (220 ms). If the participant successfully inhibited on a stop trial, inhibition was made more difficult on a subsequent stop trial by raising the ladder; if the participant did not successfully inhibit, inhibition was made easier by dropping the ladder. Moreover, when the SSD was at Ladder 4 and the participant could continue to successfully inhibit, SSD continued to increase on by 60 ms each time; when the SSD was at Ladder 1 and the participant continued to unsuccessfully inhibit, SSD would continue to decrease on by 60 ms each time. The psychophysics toolbox (http://psychtoolbox.org/) for MATLAB was used to display the stimuli.

The stop signal reaction time (SSRT) denotes the latency of the stop process and it is the most important dependent variable in the task. The SSRT can be measured from the observed distribution of RTs in no-stop signal trials in combination with the inhibition function^111,112^. In this study, we calculated SSRTs for each SSD using the integration method, and one overall SSRT was calculated by averaging all the SSRTs acquired from SSDs in our task^111–113^. ACC was also calculated: the number of correctly judged trials in go trials plus the number of successfully stopped trials in stop trials divided by the total number of trials.

#### Number–letter category switching task

The switching task was used to measure cognitive flexibility in executive functioning. This task consisted of 40 repetition trials and 40 switching trials. Each trial began with a central fixation cross for 150 ms, then a number–letter or letter–number pair was presented for 2000 ms. Participants were asked to indicate whether the number was odd or even when the letter was in red or to indicate whether the letter was a consonant or a vowel when the letter was in green. Participants were asked to respond as quickly and accurately as possible by pressing the “F” (for odd and consonant) or “J” (for even and vowel) button within 2000 ms after the onset of a stimulus. If the judgment type of the current trial remained the same as the previous one, a trial (after the first one) was defined as a repetition trial, otherwise it was a switching trial. The two conditions were presented in a pseudorandom order with no more than four repeats. The psychophysics toolbox (http://psychtoolbox.org/) for MATLAB was used to display the stimuli. The RT of this task was defined as the mean RT of switching trials minus the mean RT of repetition trials. The ACC was defined as the average of ACC in switching trials and ACC in repetition trials.

The standardized RT of the three executive functioning tasks was also averaged as an indicator to represent the ability of executive functions; the shorter the RT, the better the executive functioning. The standardized ACC of three executive function tasks was also averaged.

### Image acquisition and preprocessing

All the functional and structural data were obtained using a 3-T SIEMENS PRISMA scanner (Erlangen, Germany) at the Brain Imaging Center of Southwest University. For both task fMRI and rs-fMRI, the functional image data were obtained using a multiband T2*-sensitive gradient-recalled single-shot echo-planar imaging pulse sequence: repetition time (TR) = 2000 ms, echo time (TE) = 30 ms, flip angle (FA) = 90°, field of view (FOV) = 224 × 224 mm^2^, slices = 62, thickness = 2.0 mm, and voxel size = 2.0 × 2.0 × 2.0 mm^3^. High-resolution, three-dimensional T1-weighted structural images were obtained using a magnetization-prepared rapid acquisition gradient-echo sequence: TR = 2530 ms, TE = 2.98 ms, FA = 7°, slices = 192, FOV = 256 × 256 mm^2^, thickness = 1.0 mm, and voxel size = 0.5 × 0.5 × 1.0 mm^3^.

For BBP, preprocessing was performed in SPM12, including slice time correction, motion correction, co-registration, and affine transformation of the functional volumes to a template brain (MNI).

For EV1 and EV2 datasets, FMRIPrep^114,115^ based on Nipype^116^ was used to preprocess the functional image data with the following parameters. Each functional image data was slice-time corrected with 3dTshift (AFNI) and then resampled to their native space by applying a single, composited transform to correct for head motion. The BOLD time series were resampled to the MNI152NLin2009cAsym template. Using the implementation of Nipype (version 1.15.1), the FD was calculated for each functional image. Then, data were filtered temporally with a nonlinear high-pass filter with a 128-s cutoff.

After preprocessing, smoothing of fMRI data was performed using spatial convolution with a Gaussian kernel of 6 mm full-width half-maximum for all three datasets. We next performed fMRI denoising for all three datasets based on linear regression of the following parameters from each voxel: (a) five noise components each from minimally-eroded WM and CSF (one-node binary erosion of nodes with values above 50% in posterior probability maps), respectively, based on aCompCor procedures^117,118^; (b) 24 motion parameters (three translation, three rotation, and associated first-order derivatives and their squares); (c) all outlier frames identified within participants; and (d) linear BOLD signal trend within session. In a separate step after nuisance regression^119^, data were then temporally filtered with a bandpass of 0.008–0.09 Hz.

### Task-fMRI of AUT (EV2 dataset)

During task fMRI runs, participants were asked to think of as many novel and appropriate uses for an object in the NU condition and to think of as many GCs of an object in the GC condition. There were 11 trials under the NU condition and GC condition, respectively. These 22 trials were divided into four blocks, the first block included two NU trials and two GC trials, and blocks 2–4 all included three NU trials and three GC trials, respectively. Trials were randomly presented in each block. The objects presented in 22 trials were different (see Table S4).

Unlike previous AUT implementations, participants were required to respond verbally during the fMRI scanning. In each trial, the condition (NU/GC) was presented on the screen first; and then the cue word was continuously presented to the participants, during which time participants spoke as many novel and unusual uses of the presented cue word in the NU condition and as many GCs of the presented cue word in the GC condition. The cue word was presented for 31 s in the NU condition and 21 s in the GC condition.

The E-prime 2.0 was used to display the stimuli and to synchronize stimulus onset with MRI data acquisition. The audio was delivered via in-ear headphones. Participants’ speech was also recorded using E-prime 2.0. The Adobe Audition was used to process the recording and obtain the responses of the participants.

Five trained raters were invited to evaluate the responses of the participants for this task. The NU responses were assessed through four aspects of creativity: fluency, flexibility, novelty, and appropriateness (completely consistent with the previous “AUT”). The GC responses were assessed through three aspects: fluency, flexibility, and appropriateness. The raters were asked to score responses beyond the scoring system according to their own perception of novelty and unusualness^120,121^. An analysis of the rater agreement showed good inter-rater reliability, with Cronbach alpha scores ranging from 0.861 to 0.950.

In this study, only the novelty and appropriateness scores of the NU and image data of NU were used, and the novelty and appropriateness scores were standardized (z-transformed). Since not all participants responded in the 11 NU trials, we only reserved the participants who responded in all NU trials to ensure consistency in the number of trials (24 participants were excluded for this reason).

### Functional connectivity feature extraction

Within each dataset, we extracted the preprocessed BOLD time series from the mean across all voxels within each node defined based on previously described intrinsic functional network atlases in MNI space (Schaefer^63^ atlases of 300 cortical regions). For the EV2 dataset (task-fMRI) only, these time series were extracted for each trial based on the 31-s window prior to ideas generation onset. For both rs-fMRI datasets (BBP and EV1), we also extracted time series across the whole duration (8 min). We computed a matrix of functional connectivity values between all region pairs based on the Fisher z-transformed Pearson’s correlation coefficient of time series.

### Predictive modeling analysis of novelty and appropriateness

To examine our hypothesis at a neural level, we aimed to identify functional network-based markers of novelty and appropriateness using a CPM approach^62^ in rs-fMRI of the BBP dataset. We adopted RVR to examine the predictive performance of the functional connectome on novelty and appropriateness. RVR is a sparse kernel multiple regression method that uses Bayesian inference to obtain parsimonious solutions that generalize well and provide inference at low computational cost^122^. Notably, RVR has no algorithm-specific parameters, and no additional computational resources are required to estimate the optimal algorithm-specific parameters. By using a 10F-CV framework, participants were randomly divided into ten subsets; nine folds (90% of participants) were used as the training set, and the remaining fold (10% of participants) was used as the testing set. Within each cross-validation fold (10F-CV), we identified all node pairs (edges) exhibiting suprathreshold-level (*p* < 0.01) positive and negative correlations with novelty ratings and appropriateness ratings in the training set. We put these edges as input features into the RVR model for estimation to get a function that fits the behavior ratings (novelty and appropriateness) to the selected features, respectively. Next, the estimated RVR model was used on the test set to obtain predicted behavioral ratings (novelty and appropriateness). After all folds were completed, we obtained the predicted ratings for each participant. Because each random division results in different testing sets and training sets, we repeated the above prediction pipeline 150 times to generate 150 predicted ratings for each participant and further averaged these predicted ratings to obtain robust estimates. We finally correlated the averaged predicted ratings with the observed rating and then compared the correlation value with a null distribution.

To determine whether predicted vs observed correlations were statistically significant, we generated a distribution of null values. To do so, we repeated all of the same CPM procedures, as described, except the assignments of functional connectivity matrix were randomly permuted (1000 iterations) to obtain null correlation values to assess the significance (permutation test, *p*_pt_ ≤ 0.05). Finally, we retained edges that positively or negatively correlated with novelty and appropriateness ratings in more than 80% of the cross-validation folds to form novelty–CPM and appropriateness–CPM masks, respectively.

To avoid the arbitrariness of a single threshold, we additionally applied two common thresholds (uncorrected p values: 0.05 and 0.005) for feature selection. In addition, we also used Pearson’s partial correlation for feature selection under these three thresholds (uncorrected p values: 0.05, 0.01, and 0.005) while controlling for mean framewise head motion and common demographic variables (age, gender, and handedness). In summary, we additionally built 5 models to verify the stability of the main results model.

### The specificity of the novelty–CPM and appropriateness–CPM

To assess the specificity of model prediction in the BBP dataset, we computed the Pearson’s correlations between novelty**/**appropriateness–CPM predictions from rs-fMRI of BBP and a comprehensive battery of 60 distinct behavioral and self-report individual outcomes that were obtained in addition to novelty and appropriateness scores (see Supplementary Methods for full description). Depending on data availability, these correlations were performed with sample sizes ranging from 817 to 1447 participants (see Tables S2 and S3). However, inconsistencies in sample size caused by data matching between behavioral outcomes and functional connectivity matrixes from rs-fMRI may bring comparison errors, so we, therefore, repeated the above process using effect size (*η*^*2*^), which is not affected by sample size^68^.

We also performed the same analysis on the EV1 dataset, except that the behavioral and self-reported individual outcomes (see Supplementary Methods for full description) used were different. In addition, since the sample sizes of all data involved in the EV1 dataset are consistent, we did not additionally calculate effect sizes (*η*^*2*^).

### Analysis of functional neuroanatomical patterns contributing to the novelty–CPM and appropriateness–CPM

We visualized the edges comprising novelty–CPM and appropriateness–CPM masks (Fig. 3c) using the BioImage Suite Connectivity Visualization Tool (https://bioimagesuiteweb.github.io/webapp). To gain insight into the neuroanatomical patterns that contributed to the novelty–CPM and appropriateness–CPM, we assigned each node to 1 of 7 canonical Yeo–Krienen^69^ intrinsic functional networks (Fig. 4). For these analyses, we used the Schaefer atlas of 300 cortical regions, which includes a Yeo–Krienen network label for each node^63^. To describe our results, we use the labels provided with the publicly available Schaefer atlas (https://github.com/ThomasYeoLab/CBIG/tree/master/stable_projects/brain_parcellation/Schaefer2018_LocalGlobal).

Based on the Schaefer atlas, the novelty–CPM, and appropriateness–CPM included 296 and 430 edges, respectively, in the positive and negative masks. We assigned each of these edges to 1 of 28 within-network or between-network Yeo-Krienan pairs. To further verify the stability of the main CPM results, we also visualized the additional five models at the network level (Fig. S1). We next calculated the average correlation coefficients of the six models in the four network pairs (positive and negative network pairs of novelty–CPM; positive and negative network pairs of appropriateness–CPM). Specifically, for each network pair, we separately calculated the matrix correlation between each of the six models (a total of 15 correlation coefficients). Finally, we average these correlation coefficients for each network pair.

### Prediction of novelty and appropriateness from rs-fMRI (EV1 dataset)

In the EV1 dataset, we tested whether novelty–CPM and appropriateness–CPM predictions generalize to predictions of novelty and appropriateness from rs-fMRI data. To this end, we used the positive and the negative models from the resting-state functional connectivity data from the BBP dataset to build the predictive model for novelty and appropriateness (using the same method as the “Predictive modeling analysis of novelty and appropriateness” described). We then conducted Pearson’s partial correlation between predicted novelty**/**appropriateness ratings and observed novelty**/**appropriateness ratings, controlling for gender, age, and mean frame-wise head motion.

### Prediction of novelty and appropriateness from task-fMRI (EV2 dataset)

In the EV2 dataset, we tested whether novelty–CPM and appropriateness–CPM predictions generalize to predictions of novelty and appropriateness from task-fMRI data. We first averaged the functional connectivity matrix of each trial within each block under the NU condition (four blocks and 11 trials in total, see “Task-fMRI of AUT” for details). For each participant in the EV2 dataset, we used the positive- and the negative- models from the resting-state functional connectivity data from the BBP dataset to build the predictive model for novelty and appropriateness on each block of NU condition of AUT task-fMRI (using the same method as “Predictive modeling analysis of novelty and appropriateness” described) (refer to previous research analysis^64^ for more information). We next calculated the Spearman rank correlation between predicted novelty/appropriateness and observed novelty/appropriateness, respectively, for each participant. To generate null comparison values, we repeated all of the same CPM procedures, as described, except the assignments of functional connectivity matrix were randomly permuted (1000 iterations) to obtain null correlation values. At the group level, we performed a Wilcoxon signed-rank test to compare the within-participant prediction (predicted vs observed novelty and appropriateness ratings) vs the mean of the within-participant null correlation values.

### Mediation analysis

To test whether the novelty–CPM is relevant for associative abilities and whether the appropriateness–CPM is relevant for executive functions and associative abilities, we ran mediation analyses. The mediation analysis^123^ consisted of calculating the product of (i) the regression coefficient of the regression analysis on the independent variable (i.e., associative abilities and executive functions) to predict the mediator (i.e., novelty–CPM and appropriateness–CPM) and (ii) the regression coefficient of the regression analysis on the mediator to predict the dependent variable (i.e., novelty and appropriateness) when controlling for the independent variable. We also calculated the regression coefficient of the regression analysis on the independent variable to predict the dependent variable without controlling for the mediator (total effect) and when controlling for it (direct effect; Figs. 8 and  S3). The indirect effect was calculated as the product of Path A and Path B. All the variables entered in the mediation analyses were normalized. We tested the significance of the indirect effect using the bootstrapping method, computing unstandardized indirect effects for each 1000 bootstrapped samples, and the 95% confidence interval was computed by determining the indirect effects at the 2.5th and 97.5th percentiles. The mediation analyses were performed using the PROCESS macro^123^ in SPSS 26.0 (IBM Corp. in Armonk, NY, USA).

### Statistics and reproducibility

All statistical tests used, sample sizes, and the number of replicates are described in the corresponding methods.

### Reporting summary

Further information on research design is available in the Nature Portfolio Reporting Summary linked to this article.

### Supplementary information

Supplementary Information

Description of Additional Supplementary Files

Supplementary Data 1

Reporting summary

## Data Availability

The data and material used in this study are available from the corresponding author upon reasonable request. The source data behind the figures in the paper can be found in Supplementary Data 1.
