# Supplementary material for: Semantic associative abilities and executive control functions predict novelty and appropriateness of idea generation
Source: Commun Biol. 2024 Jun 7;7:703. doi: 10.1038/s42003-024-06405-0 (PMC11161622; doi:10.1038/s42003-024-06405-0)
Supplement: Supplementary file 1 — Supplementary Information [file 42003_2024_6405_MOESM1_ESM.pdf]

# Semantic associative abilities and executive control functions predict novelty and appropriateness of idea generation

Xueyang Wang, Qunlin Chen, Kaixiang Zhuang, Jingyi Zhang, Cheng Liu, Robert Cortes, Daniel Holzman, Li Fan, Jiangzhou Sun, Xianrui Li, Qiuyang Feng, Hong Chen, Tingyong Feng, Xu Lei, Qinghua He, Adam Green, Jiang Qiu

*Corresponding author: Jiang Qiu and Adam E. Green
**Email:** qiuj318@swu.edu.cn; aeg58@Georgetown.edu;

## This PDF file includes:

Tables S1 to S4

Figs. S1 to S3

## Supplementary Tables

**Table S1. Results of general linear models showed that associative ability and executive function independently predicted novelty and appropriateness, respectively.**

| | Model 1 | | | | Model 2 | | | |
|---|---|---|---|---|---|---|---|---|
| | *β* | *s.e.* | *t* | *p* | *β* | *s.e.* | *t* | *p* |
| Novelty | | | | | | | | |
| AA | **0.12** | **0.03** | **4.71** | **<0.001** | **0.12** | **0.03** | **4.80** | **<0.001** |
| EF | -0.02 | 0.03 | -0.69 | 0.491 | -0.02 | 0.03 | -0.89 | 0.372 |
| AA*EF | -0.01 | 0.04 | -0.24 | 0.810 | -0.01 | 0.04 | -0.28 | 0.778 |
| Appropriateness | | | | | | | | |
| AA | **-0.09** | **0.03** | **-3.35** | **<0.001** | **-0.10** | **0.03** | **-3.81** | **<0.001** |
| EF | **-0.09** | **0.04** | **-3.63** | **<0.001** | **-0.08** | **0.03** | **-3.01** | **0.003** |
| AA*EF | 0.03 | 0.04 | 1.02 | 0.310 | 0.03 | 0.04 | 1.05 | 0.292 |

In bold are correlations that reached significance after Bonferroni correction for multiple comparisons. The Model 1 included associative ability and executive function as predictors, without age, gender and handedness as additional covariates. The Model 2 included associative ability and executive function as predictors, with age, gender and handedness as additional covariates. AA, Associative ability; EF, Executive function.

**Table S2. The specific functional neuroanatomical basis for novelty derived from rs-fMRI (BBP dataset).** Novelty-CPM has the highest effect size ($\eta^2$) on observed novelty ratings (Bold at the bottom of the table).

| **Behavioral outcomes** | **Sample size** | ***r*** | $\eta^2$ |
|---|---|---|---|
| RRS_RP_Score | 1438 | -7.43E-05 | 5.520E-09 |
| NEO_C_Score | 1411 | 3.10E-03 | 9.623E-06 |
| RRS_Score | 1438 | -3.51E-03 | 1.230E-05 |
| CTQ_SFea_Score | 1438 | 4.04E-03 | 1.629E-05 |
| SWB_Score | 1438 | 4.07E-03 | 1.657E-05 |
| CFS_EC_Score | 1438 | -4.29E-03 | 1.843E-05 |
| ANT_O_RT | 1416 | -4.98E-03 | 2.475E-05 |
| CFS_AC_Score | 1438 | -6.10E-03 | 3.716E-05 |
| POMS_V_Score | 1438 | 6.40E-03 | 4.092E-05 |
| NEO_E_Score | 1411 | 6.70E-03 | 4.488E-05 |
| CTQ_SF_Score | 1438 | 7.47E-03 | 5.579E-05 |
| SRS_ES_Score | 1438 | 7.96E-03 | 6.334E-05 |
| SRS_IS_Score | 1438 | -8.27E-03 | 6.840E-05 |
| POMS_E_Score | 1438 | -8.57E-03 | 7.343E-05 |
| SRS_L_Score | 1438 | 9.23E-03 | 8.524E-05 |
| POMS_C_Score | 1438 | -1.04E-02 | 1.073E-04 |
| BIS_Score | 1446 | -1.05E-02 | 1.099E-04 |
| POMS_A_Score | 1438 | -1.09E-02 | 1.191E-04 |
| PANAS_NA_Score | 1447 | 1.10E-02 | 1.212E-04 |
| SSCS_CSE_Score | 1438 | 1.19E-02 | 1.411E-04 |
| NEO_N_Score | 1411 | 1.22E-02 | 1.495E-04 |
| CTQ_SFpa_Score | 1438 | 1.32E-02 | 1.731E-04 |
| CTQ_SFpn_Score | 1438 | -1.39E-02 | 1.931E-04 |
| PANAS_PA_Score | 1447 | 1.40E-02 | 1.948E-04 |
| RRS_B_Score | 1438 | 1.53E-02 | 2.340E-04 |
| CEI-II _Score | 1438 | 1.64E-02 | 2.701E-04 |
| SRS_F_Score | 1438 | -1.65E-02 | 2.733E-04 |
| ERQ_CR_Score | 1446 | 1.65E-02 | 2.739E-04 |
| SP_Score | 1421 | -1.67E-02 | 2.782E-04 |
| PSS_Score | 1446 | 1.72E-02 | 2.943E-04 |
| Grit_S_PE_Score | 1446 | -2.34E-02 | 5.461E-04 |
| SRS_PH_Score | 1438 | -2.47E-02 | 6.117E-04 |
| ASLEC_Score | 1446 | -2.51E-02 | 6.279E-04 |
| BIS_MI_Score | 1446 | -2.52E-02 | 6.350E-04 |
| SPQ_B_Score | 1438 | -2.60E-02 | 6.739E-04 |
| Grit_S_CI_Score | 1446 | 2.60E-02 | 6.785E-04 |
| NEO_A_Score | 1411 | -2.72E-02 | 7.385E-04 |
| SRS_PR_Score | 1438 | -2.73E-02 | 7.435E-04 |

| **Behavioral outcomes** | **Sample size** | ***r*** | ***$\eta^2$*** |
|---|---|---|---|
| ANT_A_RT | 1416 | 2.73E-02 | 7.476E-04 |
| BIS_BAS_I_Score | 1446 | -2.81E-02 | 7.885E-04 |
| SR_Score | 1421 | 2.85E-02 | 8.144E-04 |
| BIS_nPI_Score | 1446 | 2.90E-02 | 8.431E-04 |
| CD_RISC_Score | 1438 | -2.98E-02 | 8.853E-04 |
| NFC_Score | 1438 | 3.04E-02 | 9.228E-04 |
| BIS_AI_Score | 1446 | -3.06E-02 | 9.381E-04 |
| PSQI_Score | 1417 | 3.23E-02 | 1.044E-03 |
| SWLS_Score | 1438 | -3.28E-02 | 1.073E-03 |
| SDS_Score | 1438 | -3.32E-02 | 1.103E-03 |
| POMS_D_Score | 1438 | -3.40E-02 | 1.157E-03 |
| CTQ_SFen_Score | 1438 | 3.49E-02 | 1.216E-03 |
| STAI_T_Score | 817 | -3.87E-02 | 1.497E-03 |
| ERQ_ES_Score | 1446 | -4.02E-02 | 1.615E-03 |
| CTQ_SFsa_Score | 1438 | -4.32E-02 | 1.870E-03 |
| POMS_T_Score | 1438 | -4.61E-02 | 2.122E-03 |
| POMS_F_Score | 1438 | 4.74E-02 | 2.248E-03 |
| BIS_BAS_A_Score | 1446 | -4.87E-02 | 2.370E-03 |
| ANT_C_RT | 1416 | -5.69E-02 | 3.236E-03 |
| SSCS_CPI_Score | 1438 | 5.74E-02 | 3.292E-03 |
| RAPM_Score | 1417 | 7.82E-02 | 6.109E-03 |
| NEO_O_Score | 1411 | 9.88E-02 | 9.760E-03 |
| **Novelty_Score** | **1455** | **4.75E-01** | **2.256E-01** |

**Table S3. The specific functional neuroanatomical basis for appropriateness derived from rs-fMRI (BBP dataset).** Appropriateness-CPM has the highest effect size ($\eta^2$) on observed appropriateness ratings (Bold at the bottom of the table).

| **Behavioral outcomes** | **Sample size** | ***r*** | $\eta^2$ |
|---|---|---|---|
| CD_RISC_Score | 1438 | 3.51E-04 | 1.23E-07 |
| POMS_E_Score | 1438 | -3.99E-04 | 1.59E-07 |
| POMS_F_Score | 1438 | 7.87E-04 | 6.19E-07 |
| POMS_A_Score | 1438 | 1.32E-03 | 1.74E-06 |
| CTQ_SFpa_Score | 1438 | 1.81E-03 | 3.27E-06 |
| BIS_nPI_Score | 1446 | -3.73E-03 | 1.39E-05 |
| POMS_D_Score | 1438 | -4.12E-03 | 1.69E-05 |
| POMS_C_Score | 1438 | 4.20E-03 | 1.76E-05 |
| ANT_A_RT | 1416 | 4.43E-03 | 1.96E-05 |
| SRS_PR_Score | 1438 | -4.45E-03 | 1.98E-05 |
| BIS_BAS_I_Score | 1446 | 4.75E-03 | 2.26E-05 |
| NEO_N_Score | 1411 | -5.53E-03 | 3.05E-05 |
| POMS_T_Score | 1438 | 7.05E-03 | 4.98E-05 |
| ERQ_ES_Score | 1446 | -7.11E-03 | 5.06E-05 |
| CEI-II _Score | 1438 | -8.15E-03 | 6.63E-05 |
| ASLEC_Score | 1446 | -9.56E-03 | 9.14E-05 |
| POMS_V_Score | 1438 | -9.89E-03 | 9.78E-05 |
| PANAS_NA_Score | 1447 | -1.03E-02 | 1.06E-04 |
| Grit_S_PE_Score | 1446 | 1.07E-02 | 1.14E-04 |
| RAPM_Score | 1417 | -1.07E-02 | 1.14E-04 |
| SRS_F_Score | 1438 | -1.07E-02 | 1.15E-04 |
| BIS_AI_Score | 1446 | 1.07E-02 | 1.15E-04 |
| NFC_Score | 1438 | 1.07E-02 | 1.16E-04 |
| PSS_Score | 1446 | -1.10E-02 | 1.22E-04 |
| STAI_T_Score | 817 | -1.15E-02 | 1.33E-04 |
| SWLS_Score | 1438 | -1.19E-02 | 1.41E-04 |
| CTQ_SFsa_Score | 1438 | 1.24E-02 | 1.53E-04 |
| SPQ_B_Score | 1438 | -1.26E-02 | 1.59E-04 |
| SWB_Score | 1438 | 1.52E-02 | 2.30E-04 |
| CTQ_SFpn_Score | 1438 | -1.58E-02 | 2.51E-04 |
| NEO_E_Score | 1411 | -1.62E-02 | 2.62E-04 |
| SRS_IS_Score | 1438 | -1.71E-02 | 2.93E-04 |
| BIS_Score | 1446 | -1.99E-02 | 3.94E-04 |
| SRS_L_Score | 1438 | -2.06E-02 | 4.24E-04 |
| ANT_C_RT | 1416 | 2.19E-02 | 4.78E-04 |
| NEO_O_Score | 1411 | -2.24E-02 | 5.01E-04 |
| RRS_B_Score | 1438 | 2.27E-02 | 5.17E-04 |
| CTQ_SF_Score | 1438 | -2.65E-02 | 7.02E-04 |

| ANT_O_RT | 1416 | -2.73E-02 | 7.46E-04 |
|---|---|---|---|
| **Behavioral outcomes** | **Sample size** | ***r*** | ***$\eta^2$*** |
| SRS_ES_Score | 1438 | -2.83E-02 | 8.02E-04 |
| ERQ_CR_Score | 1446 | -2.92E-02 | 8.52E-04 |
| CTQ_SFea_Score | 1438 | -3.06E-02 | 9.37E-04 |
| Grit_S_CI_Score | 1446 | -3.19E-02 | 1.02E-03 |
| NEO_A_Score | 1411 | 3.20E-02 | 1.03E-03 |
| CFS_AC_Score | 1438 | -3.30E-02 | 1.09E-03 |
| NEO_C_Score | 1411 | 3.37E-02 | 1.14E-03 |
| SP_Score | 1421 | 3.51E-02 | 1.23E-03 |
| SSCS_CSE_Score | 1438 | -3.53E-02 | 1.25E-03 |
| SRS_PH_Score | 1438 | -3.62E-02 | 1.31E-03 |
| PANAS_PA_Score | 1447 | -3.65E-02 | 1.33E-03 |
| CTQ_SFen_Score | 1438 | -3.88E-02 | 1.51E-03 |
| SR_Score | 1421 | -4.35E-02 | 1.89E-03 |
| BIS_MI_Score | 1446 | -4.85E-02 | 2.35E-03 |
| BIS_BAS_A_Score | 1446 | 4.98E-02 | 2.48E-03 |
| PSQI_Score | 1417 | -5.05E-02 | 2.55E-03 |
| SDS_Score | 1438 | 5.27E-02 | 2.78E-03 |
| RRS_RP_Score | 1438 | 6.19E-02 | 3.84E-03 |
| SSCS_CPI_Score | 1438 | -6.27E-02 | 3.93E-03 |
| RRS_Score | 1438 | 6.34E-02 | 4.02E-03 |
| CFS_EC_Score | 1438 | 1.39E-01 | 1.94E-02 |
| **Appropriateness_Score** | **1455** | **5.25E-01** | **2.76E-01** |

**Table S4. Objects presented in each trial of AUT task-fMRI.**

| | | Condition | |
|---|---|---|---|
| | | NU | GC |
| Block 1 | Trial 1-4 | Coin<br>Compact disc | Ruler<br>Pillow |
| Block 2 | Trial 5-10 | Gloves<br>Eraser<br>Watermelon | Curtain<br>Writing brush<br>Comb |
| Block 3 | Trial 11-16 | Walnut<br>Hair<br>Bamboo | Clip<br>Water bottle<br>Windmill |
| Block 4 | Trial 17-22 | Calendar<br>Stapler<br>Chinese cabbage | Key<br>Fan<br>Coat hanger |

NU, Novelty Use; GC, General Characteristic.

## Supplementary Figures

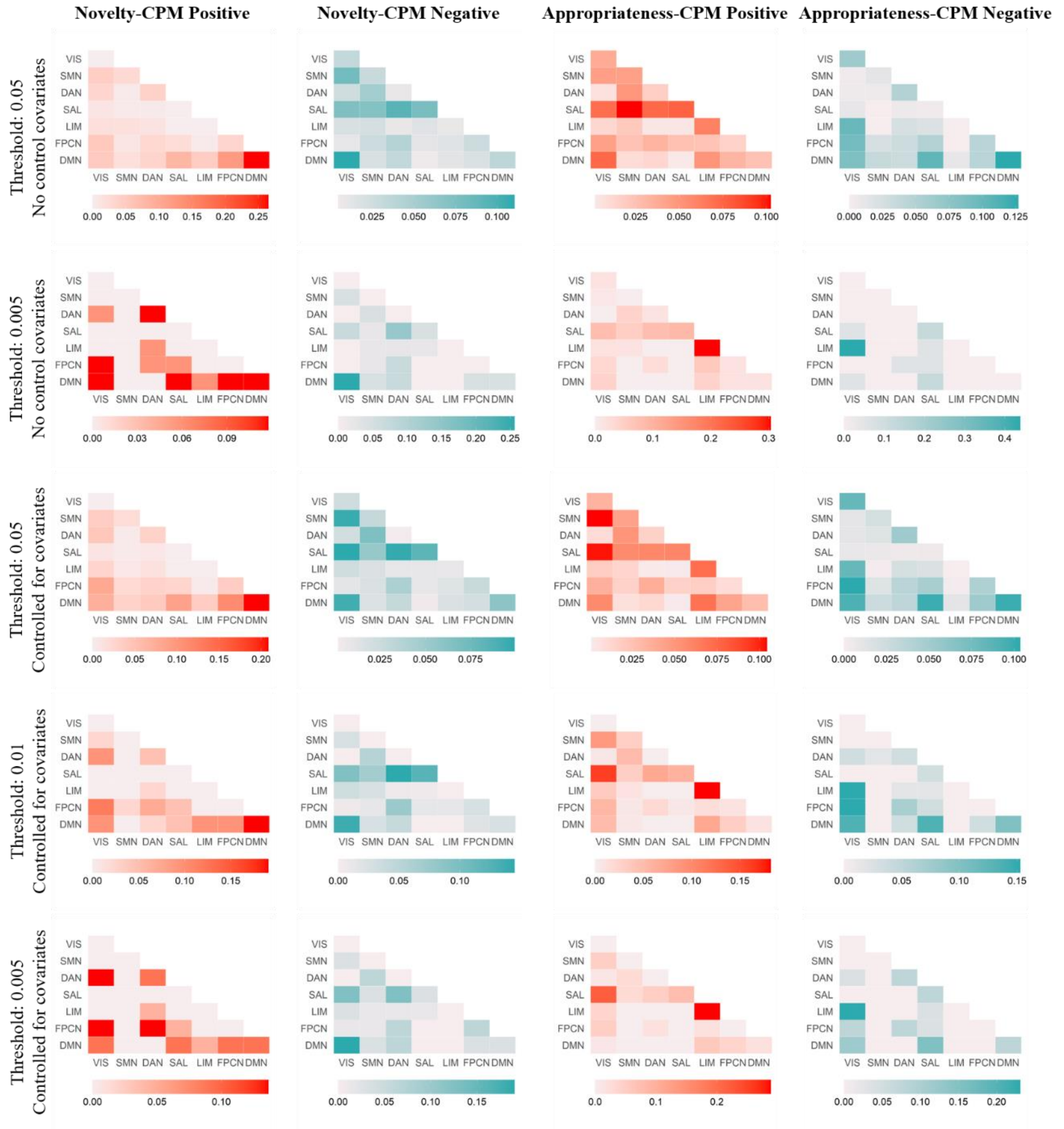


**Fig. S1. Functional neuroanatomical basis of the Novelty-CPM and Appropriateness-CPM network.** The percentage of number of edges, among those within the Novelty-CPM or Appropriateness-CPM mask, assigned to each within- or between-network pair based on the Schaefer300 and Yeo-Krienen 7-network atlases. Each column from left to right is Novelty-CPM positive mask, Novelty-CPM negative mask, Appropriateness-CPM positive mask and Appropriateness-CPM negative mask. Each row from top to bottom is the result of 5 different CPM models. DAN dorsal attention network, DMN default mode network, FCPN frontoparietal control network; LIM limbic network, SAL salience network, SMN sensorimotor network, TP temporal-parietal network, VIS visual network, CPM connectome-based predictive model.

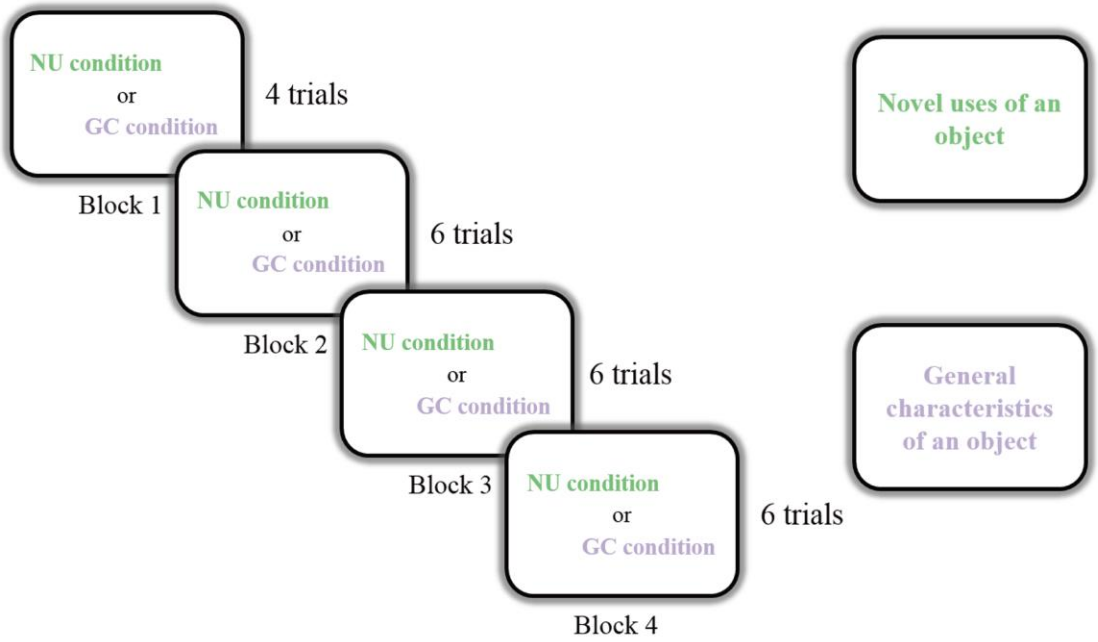


**Fig. S2. Task-fMRI of Alternative Uses Task.** NU Novelty Use; GC General Characteristic.

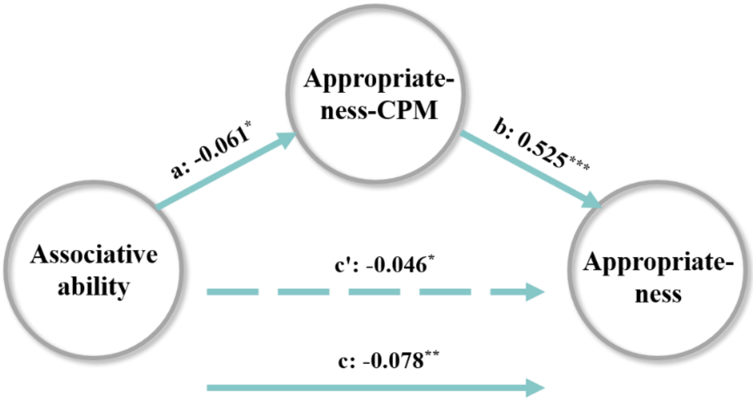


**Fig. S3. Mediation analyses.** Results of the mediation models are presented in path diagrams. Each diagram indicates the beta weights of the regression coefficients. The total effect is indicated by path c, the direct effect by path c′, and the indirect effect is given by the product of path a and path b. The mediating role of Appropriateness-CPM on the relationship between the associative ability and appropriateness ratings. *$p < 0.05$, ** $p < 0.01$, and *** $p < 0.001$.

## Supplementary Methods

### Behavioral and self-report measures (BBP dataset)

***Ruminative Responses Scale (RRS)***

The RRS contains 22 items that were rated on a 1-4 scale ('almost never' to 'almost always') [1] and was used to measure Rumination. The RRS is comprised of three subscales: reflective pondering, brooding and depression. We dismissed the depression domain due to extant research demonstrating spuriously discriminant validity with the overlapping portions across depressive symptoms [2].Thereby, the current work used reflective pondering score (RRS_RP_Score) and brooding score (RRS_B_Score), and summed them to represent the total rumination score (RRS_Score). The internal consistency of the RSS was 0.92 [3].

***Attention Network Task (ANT)***

The ANT [4,5] requires the participant to determine whether the middle arrow of 5 side-by-side arranged arrows points left or right. Participants were instructed to press "F" when middle arrow points left and to press "J" when middle arrow points right. A session consisted of a 24-trial practice block and two experimental blocks. Each experimental block consisted of 48 trials (4 cue conditions × 2 target locations × 2 target directions × 3 flanker conditions). In the present study, stimulus setup and presentation of ANT were the same as ref.[4], see ref.[4] for details of stimulus setup and presentation. RT of alerting (ANT_A_RT), orienting (ANT_O_RT) and conflict (ANT_C_RT) were used in the current work.

***State-Trait Anxiety Inventory (STAI)***

The STAI [6] consists of 20 items that assess an individual's feelings over the past week (the state scale of STAI [STAI-S]) and 20 other items that assess an individual's general feelings (the trait scale of STAI [STAI-T]) based on a 4-point Likert scale. The current research measured the trait anxiety (STAI_T_Score) of participants using STAI-T.

***Ravens Advanced Progressive Matrices (RAPM)***

The RAPM is composed of 48 nonverbal picture items. The 48 items can be subdivided in one set of 12 items (set I) and another set of 36 items (set II) [7]. Fluid intelligence was measured by set II of RAPM in the current work and participants were asked to complete it within 40 minutes. The index of fluid intelligence (RAPM_Score) was calculated as the total number of correct answers.

***Positive and negative affect scale (PANAS)***

The PANAS [8] is a self-report questionnaire that consists of 10 items to measure positive affect and another 10 items to measure negative affect. Each item is rated on a 5-point scale of 1 (not at all) to 5 (very much). The total score of positive effects (PANAS_PA_Score) and total score of negative effects (PANAS_NA_Score) were used in the current research.

***Profile of Mood States (POMS)***

The current study used the 40-item version of POMS [9]. The POMS is a psychological rating scale used to assess transient, distinct mood states based on a 5-point Likert scale (1 = not at all and 5 = extremely). The scale measures 7 different dimensions of mood swings over

a period of time. These include: tension (POMS_T_Score), depression (POMS_D_Score), fatigue (POMS_F_Score), vigor (POMS_V_Score), confusion (POMS_C_Score), anger (POMS_A_Score), and esteem-related affect (POMS_E_Score). These 7 scores were used in the current work.

***Self-rating depression scale (SDS)***

The SDS [10] contains 20 items that assess an individual's depressed feelings over the past week based on a 4-point Likert scale. The total score of SDS (SDS_Score) was used in the current research.

***Barratt Impulsiveness Scale (BIS)***

The BIS [11] is a 30-item self-report measure. Items are scored on a 4-point Likert scale from 1 (rarely/never) to 4 (almost always). Items are summed, and total scores represent total levels of impulsivity. The total score thus ranges from 30 to 120, with a higher score indicating increased impulsivity. Factor analysis revealed three independent components in the BIS[11]: attentional impulsiveness (assessing the ability to focus on the task at hand); motor impulsiveness (assessing the tendency to act on the spur of the moment); and non-planning impulsiveness (assessing the tendency to plan and think carefully). In the current research, attentional impulsiveness score (BIS_AI_Score), motor impulsiveness score (BIS_MI_Score), non-planning impulsiveness score (BIS_nPI_Score) and total score (BIS_Score) were used.

***Short Scale for Creative Self (SSCS)***

The SSCS [12] was used to measure Creative Self-Efficacy (CSE) and Creative Personal Identity (CPI). The SSCS is composed of 11 items that are scored on a 5-point Likert scale – six which measure CSE and five of which measure CPI. The score of CSE (SSCS_CSE_Score) and CPI (SSCS_CPI_Score) were used in the current research.

***Neuroticism-Extraversion-Openness Personality Inventory (NEO-PI-R)***

The NEO-PI-R [13] includes 240 items that assess 30 specific traits to define five factors: neuroticism, extraversion, openness to experience, agreeableness, and conscientiousness. Items of the NEO-PI-R are rated on a 5-point scale ranging from 1 (strongly disagree) to 5 (strongly agree). In the current research, neuroticism score (NEO_N_Score), extraversion score (NEO_E_Score), openness to experience score (NEO_O_Score), agreeableness score (NEO_A_Score), and conscientiousness score (NEO_C_Score) were used.

***Short-form Childhood Trauma Questionnaire (CTQ-SF)***

The CTQ-SF [14] consists of 25 clinical items divided into 5 subscales (5 items each): emotional abuse, physical abuse, sexual abuse, emotional neglect and physical neglect to assess these five types of childhood trauma. Each of the 25 items is a 5-point Likert-style questions, rated from 1 (never) to 5 (very often) based on the frequency of each event. Thus, total scores range from 25 to 125, while the scores of each subscale range from 5 to 25. In the current research, emotional abuse score (CTQ_SFea_Score), physical abuse score (CTQ_SFpa_Score), sexual abuse score (CTQ_SFsa_Score), emotional neglect score (CTQ_SFen_Score), physical neglect score (CTQ_SFpn_Score) and total score (CTQ_SF_Score) were used.

***Connor-Davidson Resilience Scale (CD-RISC)***
The CD-RISC [15] consists of 25 items that measure individual's psychological resilience based on a 5-point Likert scale ranging from 0 to 4. In the current research, total score of CD-RISC (CD_RISC_Score) were used.

***Curiosity and Exploration Inventory-II (CEI-II)***
The CEI- II [16] consists of 10 items that measure individual's trait curiosity based on a 5-point Likert scale ranging from 1 to 5. In the current work, total score of CEI-II (CEI-II _Score) was used.

***Sensitivity to Punishment and Sensitivity to Reward Questionnaire (SPSRQ)***
The SPSRQ [17] is a 48 yes–no response item questionnaire which incorporates two scales: sensitivity to punishment (24 items) and sensitivity to reward (24 items). In the current research, score of sensitivity to punishment (SP_Score) and sensitivity to reward (SR_Score) were used.

***Behavioral inhibition system and Behavioral activation system scales (BIS-BAS)***
The BIS/BAS scales [18] that measure individual's behavioral inhibition system (BIS) and behavioral activation system (BAS) consist of 24 items (20 score-items and 4 fillers) on a Likert scale ranging from 1 (very unlikely) to 4 (very likely). They include four subscales: the BIS scale (7 items), the BAS Reward (BASR-4 items), the BAS Drive (BASD-5 items), and the BAS Fun seeking (BASF-4 items). BIS is measured by the total score of BIS scale and BAS is measured by the total score of BAS Reward, BAS Drive and BAS Fun seeking. We used the score of BIS (BIS_BAS_I_Score) and score of BAS (BIS_BAS_A_Score) in the current research.

***Schizotypal Personality Questionnaire–Brief (SPQ-B)***
The SPQ-B [19,20] is a 22 yes–no response item questionnaire which is used to assess individual schizotypy. This self-report measure contains three subscales: the cognitive-perceptual (positive) schizotypy dimension (8 items), the interpersonal (negative) schizotypy dimension (8 items) and the disorganized schizotypy dimension (6 items). In the current research, total score of SPQ-B (SPQ_B_Score) was used.

***Pittsburgh Sleep Quality Index (PSQI)***
The PSQI [21] is a self-report questionnaire that consists of 19 items and 5 additional questions. The 19 items are combined to form 7 sleep quality component scores, including subjective sleep quality, sleep latency, sleep duration, habitual sleep efficiency, sleep disturbance, sleep medication use, and daytime dysfunction. The total score of 7 components yield a global PSQI score ranging from 0 to 21. The last five questions are scored by another person (such as a bed partner or roommate); these items are typically used for clinical information and are therefore not included in the scoring. In the current research, total score of PSQI (PSQI_Score) was used.

***Adolescent Self-Rating Life Events Checklist (ASLEC)***
The ASLEC [22] is a self-assessment questionnaire consisting of 27 negative life events that may cause psychological reactions in adolescents. Participants were instructed to first

determine whether the event occurred within a limited time or not (1 = did not occur; 2 = more than 12 months ago; 3 = within the past 12 months; 4 = currently ongoing) and rate the psychological impact of the life event (1 = no effect; 2 = mild; 3 = moderate; 4 = severe; 5 = extremely severe). The total score (ASLEC_Score) of 27 negative life events was used in our current research.

***Emotion Regulation Questionnaire (ERQ)***

The ERQ [23] consists of 10 self-report items that focuses on emotion-regulatory processes and strategies for emotion regulation and management based on a 7-point Likert scale (1 = strongly disagree and 7 = strongly agree). The questionnaire contains 10 items that capture two specific emotion regulation strategies, cognitive reappraisal and expressive suppression. The expressive suppression facet has four items and the cognitive reappraisal facet has six. In the current research, score of expressive suppression (ERQ_ES_Score) and cognitive reappraisal (ERQ_CR_Score) were used.

***Need for Cognition (NFC)***

The NFC [24] consists of 18 self-report items that is administered as an index of inherent cognitive motivation based on a 7-point Likert scale (1 = not at all and 7 = very much). In the current research, total score of NFC (NFC_Score) was used.

***Social relationship scales (SRS)***

The SRS [25] consists of 45 self-report items that is designed to summarize both the qualitative and quantitative aspects of a person’s network of relationships based on a 5-point Likert scale (1 = never and 5 = always). The SRS can also be used as a social support indicator. The 45 items are combined to form 6 component scores, including emotional support (SRS_ES_Score), instrumental support (SRS_IS_Score), friendship (SRS_F_Score), loneliness (SRS_L_Score), perceived rejection (SRS_PR_Score) and perceived hostility (SRS_PH_Score). These six scores were used in the current research.

***Satisfaction with life scale (SWLS)***

The SWLS [26] consists of 5 self-report items that is designed to measure global cognitive judgments of satisfaction with one's life based on a 7-point Likert scale (1 = Strongly disagree and 7 = Strongly agree). The total score (SWLS_Score) was used in the current research.

***Subjective well-being (SWB)***

The SWB [27] consisted of 9 items, 8 of which belong to the index of general affects scale (e.g., ‘how about the affective state you are experienced now?’), and the remaining one belongs to the life satisfactory questionnaire (e.g., ‘how did you satisfied with your life?’). Each item is based on a 7-point Likert scale (1 = Very dissatisfied and 7 = Very satisfied). The total score (SWB_Score) was used in the current research.

***Short grit scale (Grit-S)***

The Grit-S [28] is a self-report questionnaire that consists of 8 items based on 5-point Likert scale (1 = not at all like me and 7 = very much like me). The scale includes two dimensions (each with four items): the consistency of interest, which reflects a person's maintenance

of interest over an extended period of time; and the perseverance of effort, which reflects the tendency to keep trying in the face of failure or adversity. In the current research, score of consistency of interest (Grit_S_CI_Score) and perseverance of effort (Grit_S_PE_Score) were used.

***Coping flexibility scale (CFS)***
The CFS [29] is a 10-item assessment containing two subscales that measure evaluation coping and adaptive coping. Each subscale contains 5 items that are rated on a 4-point Likert scale (1 = very not applicable and 4 = very applicable). The score of evaluation coping (CFS_EC_Score) and score of adaptive coping (CFS_AC_Score) were used in the current research.

***Perceived stress scale (PSS)***
The PSS [30] consists of 14 self-report items that is used to measure the degree to which life in the past month has been experienced as unpredictable, uncontrollable and overwhelming on a 5-point response scale (1 = never and 5 = very often). The total score of PSS (PSS_Score) was used in the current research.

**Behavioral and self-report measures (EV1 dataset)**
***Creative Achievement Questionnaire (CAQ)***
The CAQ assesses 10 domains of creative achievements such as music and dance [31]. Each domain contains eight ranks with a score from 0 (I have no trained and recognized talent in this field) to 7 (my work is recognized nationwide), resulting in a skewed distribution, reflecting that few people have reached a high level of creative achievement [31,32]. The total score of CAQ (CAQ_S_Score) was used in the current research. The scores of each domain were used in the current research: visual arts (CAQ_Va_Score), music (CAQ_Mu_Score), creative writing (CAQ_Cw_Score), dance (CAQ_Da_Score), drama (CAQ_Dr_Score), architecture (CAQ_Ar_Score), humor (CAQ_Hu_Score), scientific discovery (CAQ_Sd_Score), invention (CAQ_In_Score) and culinary (CAQ_Cu_Score). We used the total score in 7 areas including music as an assessment of artistic creativity (CAQ_art_Score) and used the total score in 3 areas including scientific discovery as an assessment of scientific creativity (CAQ_sci_Score) [33].

***Inventory of Creative Activities and Achievements (ICAA)***
The ICAA questionnaire [34] to assess the real-life creative activities and achievements across eight different creative domains (i.e., literature, music, art and crafts, creative cooking, sport, visual arts, performing arts, and science and engineering). The creative activities score (CAct_Score) reflects the frequency in which participants engaged in various creative activities. The creative achievements score (CAch_Score) estimated the level of achievement acquired in a creative domain. The CAct_Score and CAch_Score were used in the current research.

***Williams' Creativity Assessment Packet (WCAT)***
The WCAT [35] contains 50 items (e.g. an item of imagination such as: 'If the final page of a storybook is missing, I will make up the story's ending myself'), provides scores for

imagination, curiosity, challenge and risk-taking. Participants were asked to rate how much they agreed or disagreed with each item on a six-point Likert scale ranging from strongly disagree to strongly agree. The total score of WCAT (Williams_S_Score) and the scores of each dimension were used in the current research: imagination (Williams_I_Score), curiosity (Williams_Cur_Score), challenge (Williams_Cha_Score) and risk-taking (Williams_R_Score).

**Supplementary References**


1. Nolen-Hoeksema, S. & Morrow, J. A Prospective Study of Depression and Posttraumatic Stress Symptoms After a Natural Disaster: The 1989 Loma Prieta Earthquake. *J. Pers. Soc. Psychol.* **61**, (1991).
2. Li, X. *et al.* An insula-based network mediates the relation between rumination and interoceptive sensibility in the healthy population. *J. Affect. Disord.* **299**, (2022).
3. Lackner, R. J. & Fresco, D. M. Interaction effect of brooding rumination and interoceptive awareness on depression and anxiety symptoms. *Behav. Res. Ther.* **85**, (2016).
4. Fan, J., Mccandliss, B., Sommer, T., Raz, A. & Posner, M. Testing the Efficiency and Independence of Attentional Networks. *J. Cogn. Neurosci.* **14**, 340–7 (2002).
5. Fan, J., McCandliss, B. D., Fossella, J., Flombaum, J. I. & Posner, M. I. The activation of attentional networks. *NeuroImage* **26**, 471–479 (2005).
6. Spielberger, C. D. State-Trait Anxiety Inventory. in *The Corsini Encyclopedia of Psychology* 1–1 (John Wiley & Sons, Ltd, 2010). doi:10.1002/9780470479216.corpsy0943.
7. Raven, J., Raven, J. C. & Court, J. H. *Manual for Raven's progressive matrices and vocabulary scales*. (Pearson, 1998).
8. Watson, D., Clark, L. A. & Tellegen, A. Development and validation of brief measures of positive and negative affect: the PANAS scales. *J. Pers. Soc. Psychol.* **54**, 1063–1070 (1988).
9. Grove, J. R. & Prapavessis, H. Preliminary evidence for the reliability and validity of an abbreviated Profile of Mood States. *Int. J. Sport Psychol.* **23**, 93–109 (1992).

10. Zung, W. W. A SELF-RATING DEPRESSION SCALE. *Arch. Gen. Psychiatry* **12**, 63–70 (1965).
11. Patton, J. H., Stanford, M. S. & Barratt, E. S. Factor structure of the Barratt impulsiveness scale. *J. Clin. Psychol.* **51**, 768–774 (1995).
12. Karwowski, M., Lebuda, I. & Wisniewska, E. Measuring Creative Self-efficacy and Creative Personal Identity. *J. Creat. Probl. Solving* **28**, 45–57 (2018).
13. Costa, P. & Mccrae, R. Neo PI-R professional manual. *Psychol. Assess. Resour.* **396**, (1992).
14. Bernstein, D. P. *et al.* Development and validation of a brief screening version of the Childhood Trauma Questionnaire. *Child Abuse Negl.* **27**, 169–190 (2003).
15. Connor, K. M. & Davidson, J. R. T. Development of a new resilience scale: the Connor-Davidson Resilience Scale (CD-RISC). *Depress. Anxiety* **18**, 76–82 (2003).
16. Kashdan, T. B. *et al.* The Curiosity and Exploration Inventory-II: Development, Factor Structure, and Psychometrics. *J. Res. Personal.* **43**, 987–998 (2009).
17. Torrubia, R., Ávila, C., Moltó, J. & Caseras, X. The Sensitivity to Punishment and Sensitivity to Reward Questionnaire (SPSRQ) as a measure of Gray's anxiety and impulsivity dimensions. *Personal. Individ. Differ.* **31**, 837–862 (2001).
18. Carver, C. S. & White, T. L. Behavioral inhibition, behavioral activation, and affective responses to impending reward and punishment: The BIS/BAS Scales. *J. Pers. Soc. Psychol.* **67**, 319–333 (1994).
19. Raine, A. & Benishay, D. The SPQ-B: A brief screening instrument for schizotypal personality disorder. *J. Personal. Disord.* **9**, 346–355 (1995).

20. Klein, C., Andresen, B. & Jahn, T. Erfassung der schizotypen Persönlichkeit nach DSM-III-R: Psychometrische Eigenschaften einer autorisierten deutschsprachigen Übersetzung des ‘Schizotypal Personality Questionnaire’ (SPQ) von Raine. [Psychometric assessment of the schizotypal personality according to DSM-III-R criteria: Psychometric properties of an authorized German translation of Raine’s ‘Schizotypal Personality Questionnaire’ (SPQ).]. *Diagnostica* **43**, 347–369 (1997).
21. Buysse, D. J., Reynolds, C. F., Monk, T. H., Berman, S. R. & Kupfer, D. J. The Pittsburgh Sleep Quality Index: a new instrument for psychiatric practice and research. *Psychiatry Res.* **28**, 193–213 (1989).
22. Liu, X. *et al.* The Adolescent Self-Rating Life Events Checklist and its reliability and validity. *Chin. J. Clin. Psychol.* **5**, 34–36 (1997).
23. Gross, J. J. & John, O. P. Individual differences in two emotion regulation processes: implications for affect, relationships, and well-being. *J. Pers. Soc. Psychol.* **85**, 348–362 (2003).
24. Cacioppo, J. T., Petty, R. E. & Feng Kao, C. The Efficient Assessment of Need for Cognition. *J. Pers. Assess.* **48**, 306–307 (1984).
25. Cyranowski, J. M. *et al.* Assessing Social Support, Companionship, and Distress: NIH Toolbox Adult Social Relationship Scales. *Health Psychol. Off. J. Div. Health Psychol. Am. Psychol. Assoc.* **32**, 293–301 (2013).
26. Kobau, R., Sniezek, J., Zack, M. M., Lucas, R. E. & Burns, A. Well-Being Assessment: An Evaluation of Well-Being Scales for Public Health and Population Estimates of Well-Being among US Adults. *Appl. Psychol. Health Well-Being* **2**, 272–297 (2010).
27. Campbell, A. Subjective Measures of Well-Being. *Am. Psychol.* **31**, 117–24 (1976).

28. Duckworth, A. & Quinn, P. Development and validation of the Short Grit Scale (GRIT-S). *J. Pers. Assess.* **91**, 166–74 (2009).

29. Kato, T. Development of the Coping Flexibility Scale: evidence for the coping flexibility hypothesis. *J. Couns. Psychol.* **59**, 262–273 (2012).

30. Cohen, S., Kamarck, T. & Mermelstein, R. A global measure of perceived stress. *J. Health Soc. Behav.* **24**, 385–396 (1983).

31. Carson, S. H., Peterson, J. B. & Higgins, D. M. Reliability, validity, and factor structure of the creative achievement questionnaire. *Creat. Res. J.* **17**, 37–50 (2005).

32. Kaufman, S. B. *et al.* Openness to Experience and Intellect Differentially Predict Creative Achievement in the Arts and Sciences. *J. Pers.* **84**, 248–258 (2016).

33. Shi, B., Cao, X., Chen, Q., Zhuang, K. & Qiu, J. Different brain structures associated with artistic and scientific creativity: a voxel-based morphometry study. *Sci. Rep.* **7**, 42911 (2017).

34. Diedrich, J. *et al.* Assessment of real-life creativity: The inventory of creative activities and achievements (ICAA). *Psychol. Aesthet. Creat. Arts* **12**, 304–316 (2018).

35. Williams, F. E. *Creativity assessment packet: (CAP)*. (D.O.K. Publishers, 1980).
