# Supplementary material for: Semantic associative abilities and executive control functions predict novelty and appropriateness of idea generation
Source: Commun Biol. 2024 Jun 7;7:703. doi: 10.1038/s42003-024-06405-0 (PMC11161622; doi:10.1038/s42003-024-06405-0)
Supplement: Supplementary file 2 — Description of Additional Supplementary Files [file 42003_2024_6405_MOESM2_ESM.pdf]

Description of Additional Supplementary Files

File name: Supplementary Data 1

Description: The source data behind the graphs in the paper （Fig. 2 – Fig. 8）
