# Supplementary material for: Semantic associative abilities and executive control functions predict novelty and appropriateness of idea generation
Source: Commun Biol. 2024 Jun 7;7:703. doi: 10.1038/s42003-024-06405-0 (PMC11161622; doi:10.1038/s42003-024-06405-0)
Supplement: Supplementary file 4 — Reporting summary [file 42003_2024_6405_MOESM4_ESM.pdf]

nature portfolio

Corresponding author(s): Jiang Qiu and Adam E. Green

Last updated by author(s): December 25, 2023

Reporting Summary

Nature Portfolio wishes to improve the reproducibility of the work that we publish. This form provides structure for consistency and transparency in reporting. For further information on Nature Portfolio policies, see our Editorial Policies and the Editorial Policy Checklist.

Please do not complete any field with "not applicable" or n/a. Refer to the help text for what text to use if an item is not relevant to your study.
For final submission: please carefully check your responses for accuracy; you will not be able to make changes later.

Statistics

For all statistical analyses, confirm that the following items are present in the figure legend, table legend, main text, or Methods section.

n/a	Confirmed	
☐	☒	The exact sample size (*n*) for each experimental group/condition, given as a discrete number and unit of measurement
☐	☒	A statement on whether measurements were taken from distinct samples or whether the same sample was measured repeatedly
☐	☒	The statistical test(s) used AND whether they are one- or two-sided<br>*Only common tests should be described solely by name; describe more complex techniques in the Methods section.*
☐	☒	A description of all covariates tested
☐	☒	A description of any assumptions or corrections, such as tests of normality and adjustment for multiple comparisons
☐	☒	A full description of the statistical parameters including central tendency (e.g. means) or other basic estimates (e.g. regression coefficient) AND variation (e.g. standard deviation) or associated estimates of uncertainty (e.g. confidence intervals)
☐	☒	For null hypothesis testing, the test statistic (e.g. *F*, *t*, *r*) with confidence intervals, effect sizes, degrees of freedom and *P* value noted<br>*Give P values as exact values whenever suitable.*
☒	☐	For Bayesian analysis, information on the choice of priors and Markov chain Monte Carlo settings
☒	☐	For hierarchical and complex designs, identification of the appropriate level for tests and full reporting of outcomes
☐	☒	Estimates of effect sizes (e.g. Cohen's *d*, Pearson's *r*), indicating how they were calculated

Our web collection on statistics for biologists contains articles on many of the points above.

Software and code

Policy information about availability of computer code

Data collection	Behavioral and neuroimaging data were obtained at Southwest University. The behavior data was obtained by E-prime 2.0 and Psychophysics Toolbox (http://psychtoolbox.org/) for MATLAB.<br>All the functional and structural data were obtained using a 3T SIEMENS PRISMA scanner Erlangen, Germany) at the Brain Imaging Center of Southwest University.
Data analysis	The MRI data were preprocessed using fMRIprep 1.15.1 and SPM12. The data analysis were carried out using custom MATLAB (2018a) code. Analysis scripts are available upon request to the corresponding author (J.Q.).

For manuscripts utilizing custom algorithms or software that are central to the research but not yet described in published literature, software must be made available to editors and reviewers. We strongly encourage code deposition in a community repository (e.g. GitHub). See the Nature Portfolio guidelines for submitting code & software for further information.

Data

Policy information about availability of data

All manuscripts must include a data availability statement. This statement should provide the following information, where applicable:

- Accession codes, unique identifiers, or web links for publicly available datasets
- A description of any restrictions on data availability
- For clinical datasets or third party data, please ensure that the statement adheres to our policy

All data are available upon request to the corresponding author (J.Q.).

Research involving human participants, their data, or biological material

Policy information about studies with human participants or human data. See also policy information about sex, gender (identity/presentation), and sexual orientation and race, ethnicity and racism.

Reporting on sex and gender	All participants' gender was based on self-reported sex in this study. We did not specially consider sex in the study design and did not analyze it.
Reporting on race, ethnicity, or other socially relevant groupings	All participants in this study were from China.
Population characteristics	BBP: mean age 21.1 ± 0.98 years; 981 females<br>EV1 dataset: mean age 21.7 ± 1.92 years; 37 females<br>EV2 dataset: mean age 21.8 ± 1.88 years; 24 females
Recruitment	In all datasets, participants were recruited via online and poster advertisements.
Ethics oversight	All participants provided written informed consent and received payment for their time and task participation, and the research protocol was approved by the ethics committee of the review committee of the Brain Imaging Center of Southwest University.

Note that full information on the approval of the study protocol must also be provided in the manuscript.

Field-specific reporting

Please select the one below that is the best fit for your research. If you are not sure, read the appropriate sections before making your selection.

☒ Life sciences ☐ Behavioural & social sciences ☐ Ecological, evolutionary & environmental sciences

For a reference copy of the document with all sections, see nature.com/documents/nr-reporting-summary-flat.pdf

Life sciences study design

All studies must disclose on these points even when the disclosure is negative.

Sample size	Final samples:<br>BBP for behavior analysis: n = 1509<br>BBP for fMRI analysis: n = 1455<br>EV1 dataset: n = 46<br>EV2 dataset: n = 31
Data exclusions	BBP for behavior analysis: 16 participants with outliers (defined as z-scores of all values of behavioral tests exceeding 3 or −3) were excluded<br>BBP for fMRI analysis: 92 participants with mean overall frame-wise displacement (FD) of >0.20 mm (based on the Power method) during rs-fMRI were excluded<br>EV2 dataset: 24 participants were excluded due to not meeting the analysis conditions
Replication	Out-of-sample validation was performed in 2 datasets
Randomization	There was no group allocation in this study.
Blinding	There was no group allocation in this study.

Reporting for specific materials, systems and methods

We require information from authors about some types of materials, experimental systems and methods used in many studies. Here, indicate whether each material, system or method listed is relevant to your study. If you are not sure if a list item applies to your research, read the appropriate section before selecting a response.

Materials & experimental systems

n/a	Involved in the study
☒	☐ Antibodies
☒	☐ Eukaryotic cell lines
☒	☐ Palaeontology and archaeology
☒	☐ Animals and other organisms
☒	☐ Clinical data
☒	☐ Dual use research of concern
☒	☐ Plants

Methods

n/a	Involved in the study
☒	☐ ChIP-seq
☒	☐ Flow cytometry
☐	☒ MRI-based neuroimaging

Magnetic resonance imaging

Experimental design

Design type: Task and resting state

Design specifications: 22 trials (Alternate Uses Task) per participant in EV2 dataset

Behavioral performance measures: BBP dataset: Self-report; Reaction time variability (computed as absolute deviation from mean for each trial)

Imaging type(s): Functional and structural (all datasets)

Field strength: 3 Tesla (all datasets)

Sequence & imaging parameters: All dataset:
Functional: repetition time (TR) = 2000 ms, echo time (TE) = 30 ms, flip angle (FA) = 90°, field of view (FOV) = 224 × 224 mm2, slices = 62, thickness = 2.0 mm, and voxel size = 2.0 × 2.0 × 2.0 mm^3.
Structural: TR = 2530 ms, TE = 2.98 ms, FA = 7°, slices = 192, FOV = 256 × 256 mm2, thickness = 1.0 mm, and voxel size = 0.5 × 0.5 × 1.0 mm^3.

Area of acquisition: Whole-Brain

Diffusion MRI: ☐ Used ☒ Not used

Preprocessing

Preprocessing software: fMRIprep 1.15.1 and SPM12

Normalization: Linear realignment to MNI152Nlin6Asym spaces

Normalization template: MNI standard space

Noise and artifact removal: 5 noise components from WM and CSF, 24 motion parameters and outlier frames

Volume censoring: No volume was discarded.

Statistical modeling & inference

Model type and settings: Connectome predictive modeling.

Effect(s) tested: Correlation between observed and predicted scores.

Specify type of analysis: ☒ Whole brain ☐ ROI-based ☐ Both

Statistic type for inference (See Eklund et al. 2016): Pearson correlation.

Correction: Family Wise Error (FWE) for behavior analysis.

Models & analysis

n/a	Involved in the study
☐	☒ Functional and/or effective connectivity
☒	☐ Graph analysis
☐	☒ Multivariate modeling or predictive analysis

Functional and/or effective connectivity: Pearson correlation.

Multivariate modeling and predictive analysis: We adopted relevance vector regression (RVR) to examine the predictive performance of the functional connectome on novelty and appropriateness. Within each cross-validation fold (10-fold cross-validation), we respectively identified all node pairs (edges) exhibiting suprathreshold-level ($p < 0.01$) positive and negative correlations with novelty ratings and appropriateness ratings in training set

This checklist template is licensed under a Creative Commons Attribution 4.0 International License, which permits use, sharing, adaptation, distribution and reproduction in any medium or format, as long as you give appropriate credit to the original author(s) and the source, provide a link to the Creative Commons license, and indicate if changes were made. The images or other third party material in this article are included in the article's Creative Commons license, unless indicated otherwise in a credit line to the material. If material is not included in the article's Creative Commons license and your intended use is not permitted by statutory regulation or exceeds the permitted use, you will need to obtain permission directly from the copyright holder. To view a copy of this license, visit http://creativecommons.org/licenses/by/4.0/
